# Enhancing Enrollment and Adherence in Long-Term Wearable Research on Dementia: Qualitative Systematic Review and Meta-Synthesis

**DOI:** 10.2196/63768

**Published:** 2025-07-31

**Authors:** Colleen M Peterson, Renée M St Louis, Carol Flannagan

**Affiliations:** 1 University of Michigan Transportation Research Institute Ann Arbor, MI United States

**Keywords:** dementia, dementia care, Alzheimer disease, caregivers, cognitive impairment, wearable electronic devices, systematic review

## Abstract

**Background:**

With the rapid expansion of wearable technologies, there is increased interest in their utility for passive data collection applications in research on aging. Wearables can be beneficial for research with people with dementia and their families, who have burdens that can make both study participation and reliable data collection more difficult, especially as dementia progresses, but their use also has challenges. Population-specific issues affecting the success of wearables for data collection can include remembering to wear a device, fluctuating acceptance of the device or study participation, and reliance on already strained caregivers.

**Objective:**

This study aimed to systematically evaluate contemporary wearables research to describe persons with dementia’s experiences with wearables, their desired qualities, and protocol needs to enhance participant buy-in and sustained wearing for better quality dementia research.

**Methods:**

We used the PRISMA (Preferred Reporting Items for Systematic Reviews and Meta-Analyses) 2020 checklist for systematic reviews and searched 3 scholarly databases using Medical Subject Headings (MeSH) terms for papers published since 2018 featuring the use or discussion of wearable devices for persons with dementia. We screened 1757 abstracts and retained 58 for full-text review.

**Results:**

We present synthesized preferences, barriers, and facilitators to buy-in and adherence to wearables in dementia research. A total of 29 factors were categorized into 4 overarching categories aligned with study development: device selection, protocol considerations, enhancing recruitment, and promoting adherence.

**Conclusions:**

These findings inform researcher guidelines for wearable device selection and protocol design to enhance the utility of wearables in future longitudinal research featuring persons with dementia and their caregivers.

## Introduction

### Background

The growing prevalence of dementia and its substantial economic and social impacts have led to increased attention and funding allocations for research supporting persons living with dementia and their caregivers as well as dementia prevention research [1]. Obtaining the perspective of persons living with dementia and their caregivers is critical to advancing our understanding of dementia to not only find effective treatments but also to address burdens often associated with providing care to this population [2,3]. Engaging persons living with dementia and their caregivers in research studies can be challenging for many reasons, including the fluctuating cognitive capacities of individuals with dementia, the logistical and emotional demands placed on caregivers, and the need for research protocols that accommodate the specific needs and limitations of this population [3]. Innovative methods to increase meaningful engagement in research studies involving persons living with dementia and their caregivers are warranted.

### Meeting Researcher Needs

Wearable technology is a noninvasive, passive way to collect a variety of data over long periods and offers the potential benefit of enhancing care and prevention efforts [4-6]. Remote data collection, such as that offered by wearable technology, has many potential benefits, including reducing recall bias and decreasing the burden placed on participants owing to frequent contact for research purposes. Identifying wearables and research protocols that meet both researcher and participant needs will help wearable technology be more effectively used in longitudinal dementia studies [7]. As the wearable technology market is expected to continue growing substantially [8], guidance for researchers to optimize the selection of devices to enhance buy-in and adherence in their studies is necessary. Wearable devices and research strategies tailored to persons living with dementia and their care providers are crucial for optimizing long-term research with this population [9].

Currently, longitudinal dementia research with wearables faces 3 overarching challenges. First, the challenges particular to persons with dementia and their caregivers and health care professionals must be considered to gain long-term buy-in and use, especially as dementia progresses and caregiving burdens increase [10,11]. Population-specific issues include remembering to wear or charge the device, ethical informed consent [12], and privacy concerns [13,14] that may differ from cognitively intact older adults [15,16]. Second, there is a multitude of wearables on the market with a variety of features and hardware forms at various costs that have not been evaluated in terms of this population’s usability and adherence factors. Third, wearables must also provide consistent, accurate, and useful data that meet researcher needs (eg, activity monitors, GPS, and location information) and have limited or easily resolvable technical problems, user-related issues, or data access factors [17].

To date, there has not been a systematic evaluation of wearables for research that holistically examines whether wearables meet the needs of persons with dementia, their caregivers, and researchers to enhance participant buy-in and research utility. Moreover, with the rapid expansion and advancement of wearable technology in the last few years, new research on participant experiences and desires for wearables is necessary. Recent wearables research with this population has featured focus group discussions with limited representation of persons with dementia and their caregivers [10,18], as compared to the experiences of older adults without dementia [19]. Observational research that could inform wearable acceptance and adherence strategies has often been limited to very short-term evaluation periods (typically up to 7 days) with a handful of activity monitoring devices [20-22]. Identifying factors related to wearables that are acceptable to persons living with dementia and their care providers, as well as population-specific support systems, is necessary to strengthen the quality of research requiring long-term adherence [7].

### Objectives

This study aimed to conduct a systematic review of dementia-related preferences, barriers, and facilitators to using wearables in research studies to inform future study development, including device selection and participant support protocols.

## Methods

### Data Sources

This systematic review followed the PRISMA (Preferred Reporting Items for Systematic Reviews and Meta-Analyses) 2020 checklist (Multimedia Appendix 1) [23]. Original research studies were identified from the PubMed, MEDLINE, Scopus, and CINAHL databases (all last consulted in January 2023) and Google Scholar. In March 2024, approximately 500 results were reviewed to capture additional references since the 2023 search. The search strategy included a combination of Medical Subject Headings (MeSH) terms and keywords. Categorized according to the PICO (population, intervention, comparison, and outcomes) framework, search terms were used in OR statements in combination for the population (“dementia,” “Alzheimer* Disease,” “memory loss,” and “cognitive impairment”) and the intervention or indicator (“wearable*,” “wearable sensor*,” “tracker*,” “biosensor*,” “Fitbit,” “wearable electronic*,” “remote monitoring,” and “pedometer”). Regarding comparison, the inclusion criteria did not require intervention trials, whereas for outcomes, the inclusion criteria did not specify the outcomes of the studies.

The full text of the relevant articles was retrieved from the Web of Science, Google Scholar, and library access at the University of Michigan. The articles were reviewed for additional related references.

### Study Selection

The selection criteria for the literature search included (1) full-text data from primary peer-reviewed journal articles, commentaries, and conference proceedings; (2) use of remote monitoring via electronic devices that captured data of persons; (3) enrollment of, or reference to, populations living with Alzheimer disease, dementias, or chronic memory impairment or loss impairment; (4) publication in English from January 2018 to March 2024; and (5) documentation of any preferences, barriers, or facilitators to wearables device use. Notably, research featuring Huntington or Parkinson disease was included if participants were experiencing dementia-type impairment and otherwise met the inclusion criteria. Studies were excluded if they (1) did not meet the inclusion criteria, (2) used an implantable wearable or a device not intended for data collection (eg, hearing aids), (3) reported only a study protocol, or (4) included no person interaction (eg, device development without consulting with the population considered affected). Dissertations, books, book chapters, other reviews, and single-case studies were excluded.

CMP searched independently, and CMP and CF reviewed titles and abstracts for relevance and subsequently conducted the full review. CMP manually searched the reference sections of these full-text articles and key reviews for additional citations. RMSL independently reviewed the search criteria and list of full-text articles to meet the eligibility criteria. The authors used EndNote (Clarivate), a bibliographic tool that allowed them to organize PDFs of articles by topic, along with other data about each article.

### Data Extraction and Analysis

Data extracted (by CMP) from the full text of the eligible studies included, when available, publication details, study location, study design, study population, device type and location worn, and device-wearing duration. For the focus of this study, we extracted information related to the preferences, barriers, and facilitators of the use, adoption, maintenance, and adherence of wearable electronic devices by persons with dementia, including ideal device qualities and protocol needs for persons living with dementia and their caregivers. This information could come from the researchers (eg, procedures and processes attempted), persons with dementia (eg, reported preferences and difficulties inhibiting ongoing use), or persons caring for those living with dementia, whether health professionals or informal family caregivers (eg, encouraging persons to wear the device).

The extracted qualitative data on preferences, barriers, and facilitators were reviewed and initially synthesized for each study by CMP into a common factor coding structure using a grounded theory qualitative research approach [24]. This approach does not presuppose findings but relies on iterative “constant” comparison of collected data to develop codes and overarching themes. RMSL reviewed the summaries and factor synthesis, which CMP and RMSL then independently assigned to each study. The factor assignments were reviewed, and discrepancies were adjudicated together. We report the descriptive frequencies of the factors identified and noted in at least 5 studies and provide a qualitative synthesis of their implications for optimizing long-term dementia research.

## Results

### Overview

**Figure 1:**
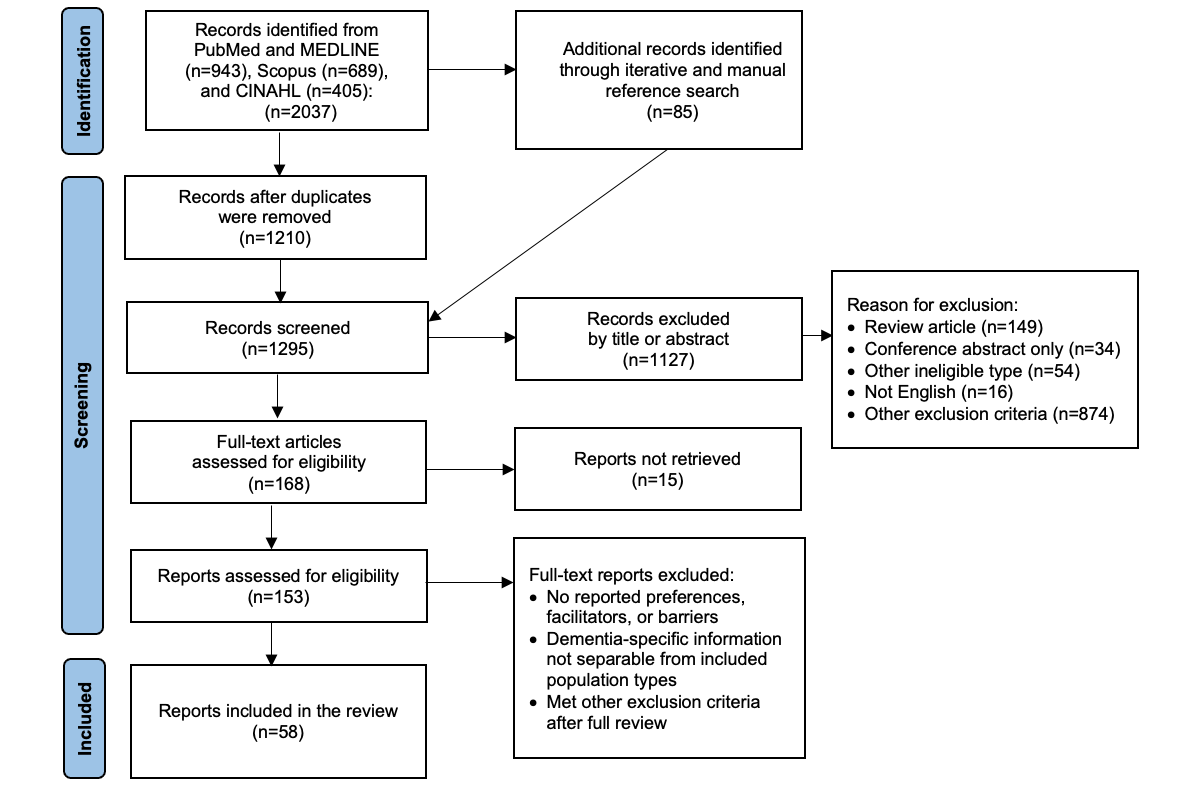
PRISMA 2020 flow diagram for the systematic review of wearables in dementia research.

This systematic review identified 1757 studies through the databases as well as iterative and manual reference searches. After the eligibility criteria were applied, 3.3% (58/1757) of the studies were retained for data extraction analysis (Figure 1 [23]). Most studies (1127/1295, 87.03%) were excluded based on the abstract review because the device evaluated was not a wearable or the population had some cognitive impairment but did not specifically include dementia. The studies were primarily observational (eg, feasibility studies) and qualitative (eg, using interviews or focus groups), with only 19% (11/58) featuring some type of trial. Participants were most often persons living with dementia in the community (as opposed to residential care), their informal family care partners, and paid dementia care health professionals. The duration for which persons living with dementia were asked to wear the study device ranged from a few hours over several days to 12 months, though 7 days was a common wear period. Information pertaining to the individual studies, including the type of wearable, is summarized in Multimedia Appendix 2 [9,10,18,20,22,25-60].

Barriers, facilitators, and preferences from the 58 studies were qualitatively synthesized into a total of 29 factors that fit into 4 overarching themes. These themes are categorized by issues dementia researchers should consider from study development to deployment: device selection, protocol considerations, enhancing recruitment, and promoting adherence. Although we categorized these factors by greatest relevance to a particular study section, it should be recognized that many of the factors are interconnected and related. The frequency and proportion of studies featuring each factor are detailed in Table 1. The factors present in each study and their cross-reference numbers are available in Multimedia Appendix 3 [9,10,18,20,22,25-77]. As we describe the factors in the results, we use the term caregivers inclusively to mean both informal (ie, unpaid, often family members) and paid caregivers (eg, health professionals and residential care staff) and provide relevant quotes that capture participant experiences. In the Discussion section, we offer a synthesis of recommendations for researchers planning to conduct longitudinal research with populations with dementia.

**Table 1:** Presence of synthesized factors across the studies (N=58).

Factor		Count, n (%)
**Device selection**		
	Easy to use	21 (36)
	Smaller size or weight	18 (31)
	Comfort	15 (26)
	Fits into routine	13 (22)
	Unobtrusive	12 (21)
	Tailorable	12 (21)
	Materials	10 (17)
	Aesthetics	8 (14)
	Easy to wear	7 (12)
	Water resistant	7 (12)
**Protocol considerations**		
	Privacy concerns	15 (26)
	Consent capacity	14 (24)
	Battery	13 (22)
	Adjustment period	10 (17)
	Task requirements	9 (16)
**Enhancing recruitment**		
	Technical anxiety	17 (29)
	Multifunctional	16 (28)
	Burden impact	16 (28)
	Stigma	13 (22)
	Caregiver buy-in	12 (21)
	Connectivity	10 (17)
**Promoting adherence**		
	Remote monitoring	19 (33)
	Provide health insight	18 (31)
	Technical support	17 (29)
	Caregiver support	14 (24)
	Safety	14 (24)
	Remembering the device	12 (21)
	Independence	10 (17)
	Self-removal	7 (12)

### Device Selection

#### Easy to Use

Given the care burden of family members or paid caregivers in residential settings, a wearable’s ease of use was paramount when asking caregivers to be involved in a study. It was the most frequently identified factor in studies, with 36% (21/58) of studies mentioning it. The wearable device must be easy to use [25-27,61-65] with an intuitive interface and features [28-31]. The device should require little maintenance or technical support for everyday use, and any interaction with the device required by the study should be simple [10,28,32-35,66]. Some studies explicitly noted that perceived ease of use was associated with more positive attitudes toward wearing the device and less effort or sense of inconvenience in participation [27,36-39]. As a person living with dementia exclaimed in the study by Gris et al [38], “Older people need to use simple things!”

#### Smaller Size and Weight

Almost without exception, participants desired smaller and lighter wearables or expressed discontent about the size and weight of the device they were asked to wear in the study [34,35,40-42,63,64,66-68]. The influence of a wearable’s size and weight was the most frequent physical form factor, with 31% (18/58) of the studies including comments about it, and it was the fourth most common factor identified overall. While rare, there were some exceptions to participants desiring smaller devices overall. Some suggested enlarging certain facets of a device, such as having a larger face or font to read [43] or larger buttons to interact with [27].

#### Comfort

The physical ease of wearing the device was important [28,31-33,38,41,44,67,69], with discomfort specifically cited as a reason why participants removed the device in several studies [34,45,66]. *Comfort* was sometimes explicitly linked with *unobtrusive*, as complementary qualities [20,29,61]*.* Nevertheless, prototype devices that were bulky or wearables with a more invasive application (eg, required taping to limbs or backs) were mostly tolerated in the short term. However, simply put, “If residents would show discomfort related to the wearable, the wearable would not be used” [61].

#### Fits Into Routine

Studies found that if a device type was already familiar or similar to what caregivers and persons living with dementia already used regularly, the wearable could be seen as convenient and more easily fit into a participant’s lifestyle [10,30,32,33,46,67,70]. Wristwatches were the most frequently cited as meeting these criteria [20,29,35,38,41,47]. At the same time, devices might also be removed if accessory removal is also part of a participant’s routine [20,32,41]: “To tackle such barriers [to using mHealth devices] will likewise require (informal) caregivers to change their attitudes towards using digital technologies and the integration of these technologies into routine care” [30].

#### Unobtrusive

A wearable that is not obvious or can be hidden was considered a distinct positive [25,29,35,38,70,71]. Having the ability to conceal a wearable alleviated some worry about a person living with dementia being resistant to wearing the device or removing it (refer to the Self-Removal subsection), which could be especially pertinent in studies involving individuals with more advanced stages of dementia [61,66,68]. Unobtrusive devices were also seen as reducing the potential of drawing attention to themselves or experiencing stigma associated with wearing a device [10,42,61] (refer to the Stigma subsection). Like the *fits into routine* factor, wristwatches were commonly cited as an unobtrusive solution [10,20], but something that could be slipped onto a belt or into a shoe was also noted as a viable option [10,29].

#### Tailorable

Having the option to use a wearable in a way that fits a person’s particular style preferences was desired, from format (eg, neck pendant or watch) to style (eg, colors and design) [10,29,34,35,38,61,62,71], as some had concerns with the particular format that was provided in the study and desired a different way to wear the device [26,31,68]. Alternative device forms can include a spectrum of participants and their day-to-day presentations (eg, gender and culture) [10,18]. Offering a wearable in several different forms could help a device suit the *fits into routine* factor, but the *tailorable* factor highlights that singular forms may not fit into everyone’s routine equally: “Several members in all four groups expressed a desire for more options in device styles...all groups asked if alternative styles could include a broach, belt clip, small bracelet, or ankle bracelet” [31].

#### Materials

Nonspecific material concerns or durability were cited as important considerations for wearables to be satisfactory to participants and researchers alike [29,41,63]. Specific material issues such as softness [34,63,66] and the need to make wearables safe for those with skin sensitivities or allergies were also mentioned [10,28,34,61]. Some participants stopped wearing the device altogether because it caused irritation [22] or even skin abrasions [48].

#### Aesthetics

The general appearance of the device design in its look and feel could be a barrier to everyday use and, thus, adherence [41,43,47,63,64]. Size, form, color, and device material all play a role in the aesthetic attractiveness of a wearable [31,34,35]. For example, participants in the study conducted by Jacklin et al [31] were dissatisfied with the prototype wearable they were testing, describing it as “big and clumsy looking” and “rubbery.”

#### Easy to Wear

Diminishing fine motor skills in persons living with dementia made *easy to wear,* or how simply one can put on or take off the wearable, an issue for day-to-day use [10,62,65]. Wearables that were difficult for the wearer to put on or take off by themselves required caregiver support that could add stress to both the person living with dementia and their caregiver, creating participation burden and a sense of lack of independence [34,48]. Some wearables were slightly more complicated and required specific sensor placement, which was either difficult to apply correctly or failed to remain securely attached [34,49,50].

The factor *easy to wear* is distinct from *easy to use* and may not play a role if a wearable is designed to be worn continuously. For example, a family caregiver reported that an accelerometer was “a little bit difficult to put on, so [the persons living with dementia] did do it but...it’s not easy.” The researchers echoed that “the wristband of the accelerometers could be difficult to fasten” but reported that afterward, “the accelerometers were not burdensome to wear” [65].

#### Water Resistant

Water-resistant or waterproof wearables were recommended [28,61,64] to minimize potential disruptions to data collection. Along with charging the device, a common point of noncompliance occurred when participants forgot to put the wearable back on after removing it for bathing (refer to the Remembering the Device subsection) [29,34]. By selecting a waterproof device and asking participants not to remove it, study protocols do not have to rely on persons living with dementia or their caregivers remembering to take off the device during bathing and, therefore, can reduce the number of study *task requirements* and related *burden impact* [44,63]. Cruz-Sandoval et al [29] suggested that minimizing the need to remove a device can reduce instances of “resistance” from persons living with dementia, and therefore, waterproof devices “can ease some of the burden on caregivers” as well.

### Protocol Considerations

#### Privacy Concerns

Many studies noted that privacy was an issue, with participants voicing concerns about data security and who had access to the wearable’s data [10,18,26,31,38,63,68,71]. For example, a caregiver’s spouse worried about third-party breaches of her wearable data, explaining that “privacy and security are important to his wife because ‘...she understands just how easily those systems can be hacked’” [71].

At the same time, some persons living with dementia and their caregivers stated explicitly that privacy concerns were outweighed by the personal or societal benefits of their study participation [25,33], or they reported no privacy concerns [27]. Participants understood that data sharing was often necessary to meet the goals of the study, and privacy concerns were ameliorated when researchers established trust around data management: “Both informal caregivers and the nursing staff were not concerned about privacy issues regarding the wearable, as they consider biomedical variables as de-individualized values. The participants trusted the researchers and nursing home regarding good data handling, as they felt well-informed” [61].

Researchers were able to build trust and attenuate privacy concerns through transparency about data access and security measures during the informed consent processes and throughout the study [61,72]. Empowering participants to have control over privacy options (eg, allowing the collection of some but not all data) and offering the ability to turn off or select what data to monitor also helped [51,73].

#### Consent Capacity

The ability of persons living with dementia to understand and actively affirm their study participation is an ethical imperative but was specifically mentioned as a potential difficulty by several studies [38,71]. Especially in longer-term studies, seeking ongoing consent to participate was highlighted as necessary, with suggestions to check in regularly to assess the cognitive capacity of the persons living with dementia and their willingness to participate [20,51,74]: “Fluctuating cognition can be a significant barrier to the remote monitoring of isolated patients as forgetting about one’s motivations to be monitored can lead to anxiety and agitation for being watched and, consequently, the disablement or destruction of equipment” [74].

At odds with fully informed consent was the general awareness of persons living with dementia regarding their participation or the device [32,43]. At this level of impairment, consent from a caregiver or another proxy for the persons living with dementia was sometimes sought instead, which could be an appropriate barrier to inclusion [22,69,70]. Another major challenge is reconciling what to do when a person living with dementia desires conflict with the caregiver’s desire for them to engage in the study and wear the device [10,68]. Under consideration is the issue of dignity, respect for autonomy, and how ignoring these or not obtaining assent could lead to agitation: “In one case, [the study staff] visited the participant after he had refused to wear it on his wrist, saying his wife was tagging him ‘like a dog’” [68]. In another study [10], a caregiver reported “[My husband] would have never given his consent even at the beginning—he liked the independence of making his own decisions and doing what he wanted. And I would have had to do it without his consent.”

It was recommended that, when possible, researchers, caregivers of persons living with dementia, and their proxies should attend to their care recipient’s wishes, whether from an explicit advanced directive [73] or knowledge of their comfort with similar technology [30].

#### Battery

The longevity of battery life and the ease of charging were noted as concerns for both researchers and participants [9,28,38,63,64,75]. Wearables with a battery life of at least several days were ideal [28,29,52,63], decreasing the need for participants to remember to charge the device [9,10,72] (ie, simplifying *task requirements*), which also minimized data collection interruptions for researchers. Low battery warnings from the device or study staff were seen as a useful tool to ensure an adequate charge of the battery [10,32,68]. Necessarily, the burden of charging was exacerbated when a caregiver was not on site to physically assist or when the charging mechanism was difficult to implement [35].

#### Adjustment Period

Participants may need time to acclimate to wearing the device (eg, establish wearing routines) and to resolve technical issues before consistent adherence and, therefore, consistent data quality, as adherence tended to improve after an initial period [9,29,32,33,41,53,61]. On the other hand, some studies found better compliance earlier in a study period [42,54,65], perhaps because of novelty. Adjustment periods featuring cognitive aids such as instructional pamphlets and study staff check-ins provided early troubleshooting and time to build new routines [9,29,33].

#### Task Requirements

Cognitive impairment and caregiving burden were barriers to protocol adherence when participants were asked to do more than simply wear the device [55,56,74,75]. When a study protocol required additional activity from either the persons living with dementia or a caregiver, such as completing bedtime and wake-up event markers, they were less likely to remember or be able to comply [9,50,57,72].

### Enhancing Recruitment

#### Technical Anxiety

Although many interacted with the study wearables and related technology platforms successfully, participants’ sense of a lack of technical proficiency and negative attitudes about technology were reported as a perceived barrier to enrollment [10,18,27,37,38,41,71] and adherence during the study [29,53,62]. Caregivers and persons living with dementia alike expressed concern about the complexity or technical know-how needed to operate the wearables [29,36,53,63,74]. Technical anxiety could present as worrying about damaging or losing the device [20,29,34]. The level of technical anxiety and attitudes toward the technology were related to age qualitatively and statistically [10,27,38,53,62] and previous experience with devices [36,46,76]. Participants in a study actually rated themselves as less technologically proficient at the end of the study because of difficulty with the wearable [42].

#### Multifunctional

Participants saw the potential for the device and platform to offer more information or other uses to the wearer as a benefit of being involved in the research [27,71]. Being able to track their health and activity, signal health events or emergencies (eg, wandering or fall alerts), provide directions, or offer location services, among others, were cited as desirable features [10,25,27,29-31,34,63,64,67,74]. Simpler functions such as information about time, date, and alarm capacity were also ideal wearable facets [10,32,35,41,52,63]: “Participants with dementia indicated that multiple purposes for the locator device, such as being able to measure heart rate, blood pressure and activity level, is advantageous as it would streamline the number of devices that they and their caregivers must contend with” [10]. Stavropoulos et al [63] reported, “Feedback suggested that some people with dementia may be more willing to accept technology that supports them in their daily functioning, in addition to assessing it."

#### Burden Impact

Caregivers especially liked the potential the wearables offered in terms of supporting their caregiving role and tasks, which, in turn, reduced related burden and stress [27,32,39,64,69]. Factors such as a wearable’s capacity for *remote monitoring* and *providing health insights* could improve quality of life or provide peace of mind [33,39,63,67,72]. However, when the wearable was technically difficult to use (*easy to use)* or researchers required additional time or study activity (refer to the Task Requirements subsection), being involved in the study was perceived as a cause of additional burden and a deterrent to both enrollment and continued participation [29,32,33,58,59,61,70,74]. Notably, participants in several studies reported experiencing a mix of both reduced and heightened burden, which varied according to specific facets of the research or from using the wearable [32,33,65,69]: “The nursing staff indicated that they did not want to spend valuable care-time if the foreseen benefit of the wearable for the specific resident was not evident to them. Likewise, also informal caregivers found it important to know the potential individual benefits, before deciding whether to bother the resident with the wearable, as they anticipated some level of resistance to wearing the wristband by the residents” [61].

#### Stigma

Caregivers and persons living with dementia expressed concerns about the stigma associated with the wearable and the wearable identifying their care recipient (or themselves) as affected by the hidden disability [10,18,30,64]. There was concern about social acceptance and the visibility of the device, as well as the perceptions or comments that it might engender (eg, that they are under surveillance or need to be monitored) [33,34,61,72]. On the other hand, “social influence” was often a positive, with care providers or fellow persons living with dementia offering positive comments or encouraging the use of the wearable [29,37,39,76].

Families and professional care providers alike were sensitive to the negative connotation that some wearables might cause. An Alzheimer Society staff member reported that some families might avoid incorporating a MedicAlert bracelet in their care management plans because “It’s a signifier or something that’s perceived to bring stigma—[the persons living with dementia] don’t want to be out there clearly marked in some way, that there’s something wrong with them. Nor does the family” [18].

#### Caregiver Buy-In

Caregiver buy-in underlies many facets of wearable studies featuring persons living with dementia, as many studies relied on caregiver commitment and coordination with the researchers to do study activities (refer to the Caregiver Support subsection) [20,36,57,61,70]. Moreover, they played significant roles in facilitating understanding of the study by persons living with dementia [29,47] and in encouraging them to wear the device [39,41]. Some studies specifically cited the need for caregivers to have perceived usefulness or reasons to commit to reducing perceived potential burden or risks to enroll themselves and their care recipient in the study [61,72,76]. Gaining caregiver buy-in could be enhanced by reducing participation burdens (eg, fewer *task requirements*, *easy-to-use* devices, and offering ongoing *technical support*) and experiencing personal benefit from the study (eg, *remote monitoring* and *providing health insights*).

#### Connectivity

Passive, remote monitoring is the defining advantage of wearable device studies. However, technical connectivity requirements can be a barrier to enrollment [10,31,61,69,74] and may create logistical problems for data transmission [18,31,56,77]. For example, requiring faster Wi-Fi or consistent Bluetooth linkage with a smartphone for more frequent data uploads [67,75,77] may introduce recruitment bias to individuals of higher socioeconomic status and those who are already technically inclined: “The lack of internet connection in patient homes and of funding for caregivers and family member to purchase assistive or communication devices are frequently coupled with skepticism or low abilities to engage with digital products” [74].

### Promoting Adherence

#### Remote Monitoring

Remote monitoring was the most frequently cited desirable and useful feature (mentioned in 19/58, 33% of studies) when engaging in a wearable study among populations with dementia [10,25,27,34,59,61,69,76]. Geolocation-based remote monitoring enabled caregivers to track the movements of persons living with dementia when they were not present, including movement outside the home. Remote monitoring was also seen as a way to know about the well-being of persons living with dementia, for example, by the identification of agitated movement or interrupted sleep [32,40,77]. Remote monitoring enhanced the *independence* of the person living with dementia [27,38,63] while providing informal caregivers with peace of mind regarding the safety of persons living with dementia (eg, reduces *burden impact*) [46,63,64,67,72].

For some participants, the utility of the benefits of geolocation services conclusively outweighed potential *privacy concerns* [25,42]. However, for some, introducing the idea of being monitored could be seen as an infringement on autonomy and privacy [25,31], such as when “some participants feared that proud Anishinaabe older adults would be offended by the idea that their movements would be tracked” [31].

#### Provide Health Insight

Providing health insight to the participants was one of the top factors mentioned as a benefit to ongoing participation and use of a study’s wearable. Paralleling the *multifunctional* factor as an aid to enrollment incentives, health insights included, for example, information on blood pressure, heart rate, sleep quality, and steps. Having ongoing access to this health data motivated participants’ personal activity, helped caregivers monitor for health crises, and provided more general insight into the well-being of persons living with dementia [10,25,27,29,32,33,38,53,54,58,61,63,69,74,77]. In addition, they appreciated that this health information could be useful to residential staff and physicians in informing care decisions [25,27,32,34,61,64,69]. A caregiver in the study by Lazarou et al [25] said that with the information from the wearable, “we could monitor her daily activity and communicate any problems to the doctors. It helped us a lot to understand what is going on.”

#### Technical Support

As *technical anxiety* was a barrier, providing initial technical training and ongoing technical support was identified as a complementary facilitator [20,26,37-39,50,74]. Staff availability to provide training at enrollment, support during an adjustment period, and assistance as needed throughout the study helped improve adherence [29,33,77]. Stand-alone instructional materials and cognitive aids were useful to participants who sought to troubleshoot issues on their own [9,10,47,66]. Along with data monitoring, regular proactive check-ins with participants made (or would have made) staff aware of technical problems and helped maintain better compliance [29,45,53,68,77].

For example, Gelonch et al [33] provided caregivers with initial training and ongoing technical assistance, and consequently, they supported their care recipient’s use of a lifelogging camera: “The [persons living with dementia] said that the support and supervision provided by their caregiver in certain tasks or actions, such as connecting and loading the camera, were also key for them continuing with the experiment.”

Even when apprehensive about using the technology (refer to the Technical Anxiety subsection), participants commonly showed a readiness to learn how to operate the wearable devices and follow study protocol. As recounted by Anderson et al [36], “Many were not necessarily technologically proficient caregivers, but they wanted to be useful. None of the caregivers implied that the research should not continue to be refined.”

#### Caregiver Support

While the importance of *caregiver buy-in* was highlighted in recruitment, in this section, we underscore the importance of caregivers and their investment in the study for continued wearables adherence [36,39,71,77]. Many research protocols required caregiver activity to accomplish their goals by having them be the primary study participant or helping persons living with dementia complete study tasks [29,44] and, importantly, charging the wearable [35,77]. Many factors depend on the caregiver, such as helping persons living with dementia *remember the device* [20,41], assisting with putting it on (refer to the Easy to Wear subsection) [34,48], or understanding how to operate the wearable [33]. In addition, caregivers could be instrumental in explaining facets of the study and, in other ways, facilitating communication of research needs to their care recipient throughout the study [29,68].

Notably, relying on caregiver support can be a major ask (ie, increase *burden impact*): “The caregivers were able to be present almost all the time. They demonstrated personal investment in the care that the person living with dementia received. Their willingness to participate in, work with researchers, and use technology 24 hours a day for 30-60 days was a significant commitment” [36].

#### Safety

Broadly speaking, wearable technologies were seen as an instrument for enhancing the safety of persons living with dementia [45,46,71,72]. This potential positive was specifically identified as an ongoing benefit, often in tandem with or implied within *remote monitoring* and *providing health insight*. Wearables offer a sense of security to persons living with dementia and their care providers, aiding aging in place by alerting caregivers to life events or potential crises (eg, elopement or fall monitor) and detecting changes in health or behavioral patterns [28,31,34,59].

On rare occasions, the device itself was harmful, such as when an over-ear device caused skin abrasions [48] or when participants desired a different product or sensor design for safety reasons [69]. In contrast to other participants in their study, a caregiver in the study by Snyder et al [71] explained that they felt the wearable, an emergency response pendant, gave a false sense of security, saying, “My reluctance is that it doesn’t seem...It can’t stop something from happening to her. It just alerts you maybe if something does.”

#### Remembering the Device

Adherence in wearable studies was affected by persons living with dementia’s ongoing ability to remember what the device was for [22,29,42]. Not remembering the purpose of the device could also result in “confusion and agitation” [43]. More commonly, persons living with dementia simply forgot to wear the device [10,20,63,75], especially after removing it for bathing (if not *water resistant*) or sleep, often resulting in failure to put it back on [34,65]. Better adherence was reported earlier in the day [53,77], potentially because of sundowning: “Due to memory impairment participants had to be constantly reminded to wear the device and they could change their opinion from one day to the other. Activities that made wearing the device more salient, such as the need to take it off to charge it or bathing, proved challenging with some participants” [20].

#### Independence

Whether the device provided geolocating, physical activity monitoring, or health alerts, participants, especially caregivers, saw these devices and platform features as enhancing the independence and autonomy of persons living with dementia [10,27,39,63,67,71]. Participants’ ongoing access to the data and ability to turn off data collection at their discretion contributed to a sense of agency within the study itself [33,38]. Similar to the potential harms of *remote monitoring*, Synder et al [71] framed this positive as part of the “conundrum between the benefits of having peace of mind, a sense of self-efficacy, and greater independence versus perceiving (remote monitoring technologies) as an invasion of privacy, a security risk, a false sense of security, and a distraction.”

#### Self-Removal

Taking off the device when not instructed was an issue in several studies [44,60]. This could be temporary, such as during bathing, or routine, such as removing accessories before sleep [32,72]. In some cases, removal was longer term, often due to agitation (possibly related to sundowning) or discomfort [22,32,66]. Some researchers and care providers suggested or chose to put the wearable out of reach (eg, on their back) to prevent self-removal by persons living with dementia [60,61].

## Discussion

### Principal Findings

#### Overview

This review identified barriers, facilitators, and preferences that could improve the quality of research studies featuring wearable devices and persons living with dementia. The review included studies encompassing a variety of study designs and perspectives from researchers, persons living with dementia, and their caregivers (both unpaid family members and paid professionals), ensuring broad inclusion of research experiences. We offer a qualitative synthesis of these findings to enhance enrollment and adherence in long-term wearable research featuring persons living with dementia. Recommendations for future research fall within 3 main categories: device selection, ethical study design, and technical and data management plans.

#### Device Selection

##### Dementia Impairment Level Requires Different Devices

We identified the importance of selecting multifunctional devices with the capacity for participants to monitor their own or their care recipient’s data, whether on the device itself or via a dedicated platform. However, what persons living with dementia and their caregivers preferred in terms of devices was sometimes different depending on the advancement of dementia in their target population. In earlier stages, persons living with dementia themselves were more engaged and desired more interactive, multifunctional devices that provided personal benefit to them. However, persons living with more severe dementia, from their perspective and their caregivers’ perspectives, desired or interacted with only simpler wearable form factor and features (eg, just used the clock feature) [77]. Even less obtrusive devices or ones out of reach may also be warranted if they are more likely to remove the device themselves as dementia progresses.

The growing ubiquity of remote monitoring and wearable devices for everyday use likely makes stigma less of a concern as we move into the future because an older adult will not stand out when wearing something such as that, especially if it is unobtrusive or looks like an everyday wristwatch. The availability of the devices likely also drives expectations of what participants will personally gain from being in a research study, as people are more familiar with what type of information gathering is theoretically possible, and participants may expect to have access to that information too.

##### Wristwatches Fits Into Routine but Could Also Be Problematic

Wristwatch wearables were highly recommended as they are part of normal wear for many and offer different levels of multifunctionality. In addition, their unobtrusiveness can reduce stigma [10]. However, habitual device removal during sleep and bathing often conflicted with the research protocols requiring participants to wear the devices all the time [32,41]. Waterproof forms, along with clear instructions for the caregiver and persons living with dementia to avoid removal in these situations, are useful. Researchers should understand that adjustment periods may be warranted with proactive reminders as participants acclimate to the newer routine and have fewer persistent issues [41].

#### Ethical Study Design

##### Attend to Fluctuating Consent Capacity in Persons Living With Dementia

Persons living with dementia feel actively engaged in the research and empowered over their health status when they can see their health data and decide when to engage in the study. However, progressive impairment can mean a person living with dementia may experience fluctuating ability to consent and understand the protocol for a long-term study. Researchers in longer-term studies should establish ongoing communication to monitor possible changes in device preferences or needs. Advanced cognitive decline from dementia can mean decision-making and consent ability are totally compromised for the person living with dementia. Reliance on a caregiver or another surrogate for consent is common, but studies emphasized that if the person living with dementia has given their thoughts on similar situations before, those preferences should be prioritized. Respecting participants’ agency is paramount; recognizing and responding to the issues of ongoing consent in this population is key to conducting an ethical study. Actively checking in with participants on a regular basis in long-term studies to gain assent or consent for continued data collection is ethical and may also be required by institutional review boards, given expected cognitive changes.

##### Promote Inclusivity With Wearable and Protocol Choices

Researchers should be aware of how their study protocol and wearable device requirements may impact who can or would enroll in their study. Some studies, such as that by Cohen et al [53], suggested screening participants for technical knowledge and ability as a way of ensuring compliant participants are enrolled. However, this is not inclusive and could result in bias in recruitment. Instead, preexisting technology and connectivity needs (eg, stable Wi-Fi) should be minimized or eliminated to enhance distributive justice [73,78] and increase the representativeness of the study results. Researchers can reduce the burden and increase equity by not requiring constant wireless connectivity or by supplying the technology to participants. However, intermittent data collection may be at odds with proactive data monitoring for troubleshooting purposes. Staff phone calls or other lower-technology check-ins are recommended in this case.

If possible, different formats and styles (eg, watch vs pendant and watchband options) that are familiar and fit a participant’s personal, cultural, and gender preferences should be offered [79]. However, it is important to balance these preferences with the type and quality of data that each format can reliably collect [34]. As evidenced by this review and other research efforts, user-centered codevelopment of wearables with persons living with dementia for research purposes is feasible [38,80] and should continue to be explored to enhance recruitment and adherence in longitudinal studies featuring persons living with dementia.

#### Data Management

##### Implement Data Management Plans

We categorized a total of 29 factors into 4 categories that aligned with study development (device selection, protocol considerations, enhancing recruitment, and promoting adherence). However, a fifth category—data management—was largely left uncommented on, as studies were primarily focused on the needs and preferences of the participants rather than the researchers. Participants may report ongoing adherence, but making sure a wearable is not only worn but also collecting data is paramount to conducting quality longitudinal research.

Researchers’ data management plans should comprise data collection, storage, and processing for analysis [29,73]. For example, researchers conducting long-term studies should know how long their device batteries will last under their protocol and have procedures in place to limit both data loss and participant burden (eg, reminders to participants to charge their devices). By regularly checking data for completeness, researchers can identify technical problems and offer troubleshooting to resolve missing data issues. Researchers should also have a loss prevention plan for the wearable to reduce missing or inactive devices and the resulting data loss.

For example, Cruz-Sandoval et al [29] had a comprehensive data management plan for a wearable study in an assisted living setting. They combined weekly program visits with battery charging, data collection (ie, upload and synchronization with the study server), and addressed technical troubleshooting needs. They also reviewed the uploaded data for abnormal data patterns and double-checked with caregivers “as soon as possible” to understand if the abnormal data were valid or if they should flag it for deletion. As a loss prevention plan, the researchers also asked the staff to report on the location of the device at the end of each work shift. Particular data management plans could also influence device options; Muurling et al [81] recommended that real-time participant access to device data could both increase adherence and help alert researchers to data issues in close to real time.

##### Collective Data Management Efforts Could Enhance Support for Wearables Research

To reduce researcher data burden in individual studies, research groups could build infrastructure and codebases to share collectively in an open-access format (eg, on GitHub). Although researcher perspectives on the use of the devices and subsequent data management were generally missing in the studies, 1 study reported that their experience organizing, cleaning, and analyzing Fitbit data were “onerous and time consuming” and recommended that others use automated data management software [77]. Researchers should be aware that many commercial, proprietary wearables may not offer researchers access to the raw data, which may limit secondary utility and increase reliance on the original developers for measure validation. Other collective data enhancements include setting standard definitions or ideal compliance goals, which varied across studies or were not reported at all. Clear, agreed-upon definitions for adherence and reporting standards are critical for improving long-term dementia research with wearable devices [9,47]. Indeed, a lack of specific, consistent adherence and attrition data from many studies made quantitative comparisons related to the factors impossible. When combined with real-time data monitoring, shared data expertise can help researchers determine whether seemingly incoherent data result from technical issues, allowing for timely resolution [81], or from changes in cognitive function and life circumstances as dementia progresses [82].

### Informing Wearable Technology Adoption and Use Models

This systematic review evaluated preferences, barriers, and facilitators to wearables in a study setting, so everyday technology adoption measures may not be entirely applicable. Indeed, caregivers and persons living with dementia may endure adjustment periods, committing themselves to using difficult technologies if the knowledge gained will advance dementia prevention efforts or benefit other caregivers [82]. However, because of the lack of consistent quantitative data, we could not assess factor relationships quantitatively with wearable technology adoption and use models such as the unified theory of acceptance and use of technology model [83], its extended version—the unified theory of acceptance and use of technology 2 model [37], the wearable acceptance in health care model [84], or the technology acceptance model 3 [85]. These measures may also need to be updated as overall perceptions of technology change and its use becomes more ubiquitous, even among older adults. Surveys and other marketing research indicate that although fewer older adults use wearables compared to other age demographics, approximately 25% of US adults aged ≥65 were using wearables in 2023 [86], a rapid increase even in just the last few years [87]. Moreover, these measures relate to technology acceptance in general, while motivations underlying intentions and actual use of wearables may be different, especially as noted within research contexts. A research-specific technology acceptance questionnaire could help researchers understand perceptions directly related to their needs and identify additional barriers to recruitment and adherence.

There is also utility in a more systematic assessment of wearable device qualities themselves and how they relate to adherence. The use of measures such as the Quebec User Evaluation of Satisfaction With Assistive Technology (QUEST; version 2.0) can simplify device comparisons and inform future wearable choices or commercial development. Future reviews could also benefit from standard reporting of why particular devices were chosen in original studies.

### Clinical Implications

We have reviewed and identified factors related to the use of wearables in long-term dementia research. However, wearables are also a potentially viable way to gather data for clinical decision-making because they can offer a way for clinicians to proactively monitor health data and circumvent some of the challenges around gathering a patient history from someone with dementia [64,88]. Supporting this potential, a large cohort study of cognitively healthy participants showed that changes in long-term 24-hour activity patterns were associated with elevated risk for the onset of Alzheimer disease, Parkinson disease, and cognitive decline [89]. Moreover, as wearable devices are being used more by the general population, clinicians are experiencing patients coming in with questions about what they have learned from their own wearable devices. Improving research in this area can help clinicians think about how they can interpret and use wearable data in a meaningful way (eg, personalized medicine). Clinicians could use the results of this paper to guide a conversation with their patients about selecting a wearable that is beneficial for meeting the needs of both the patient and their health care team, which could also enhance adherence.

### Limitations and Strengths

Our findings are generally agnostic of cognitive impairment status or degree of dementia, reflecting the participant mix seen in the studies, which also often did not disambiguate by the severity or type of dementia (eg, Alzheimer disease and Lewy body disease), which may influence findings as well as the generalizability of findings. Because our analysis was not limited to specific device types or study goals (eg, physical activity or dementia prevention), not all factors may be applicable to all future research applications. We did not perform a formal risk of bias assessment because of the heterogeneity of the study types included, and we note that many studies may be considered at high risk of bias because of smaller sample sizes. However, considering the outcomes of interest for this study were qualitative in nature, the samples were considered sufficient.

Because our extraction captured all reported barriers and facilitators regardless of their origin, we identified the holistic perspective of researchers, persons living with dementia, and their care providers, including unpaid family members or paid health professionals. We systematically coded barriers, facilitators, and perspectives using a grounded, qualitative approach, which did not presuppose expected outcomes, allowing for new or unexpected insights. We offer a comprehensive synthesis of multiple types of studies featuring many wearables and outcomes of interest, identifying overarching themes core to long-term wearable research featuring populations with dementia.

### Conclusions

We described factors critical to enhancing dementia study recruitment and wearable adherence, emphasizing how those factors should be considered collectively in an ethical context to meet both researcher needs and participant desires. Organized by study design order and literature prevalence, dementia researchers can use the findings of this systematic review to thoughtfully design quality, long-term wearable studies. The incorporation of wearable technology in dementia research presents a promising pathway to surmount traditional challenges associated with studying persons living with dementia and their caregivers. By leveraging these noninvasive tools, researchers can obtain continuous, objective data for use in advancing treatment, care, and prevention efforts. The results of this evaluation will facilitate quality improvement in ongoing research efforts and contribute to a more person-centered approach to developing effective interventions and prevention strategies.

## Appendix

Multimedia Appendix 1 PRISMA 2020 checklist.

Multimedia Appendix 2 Summary of included wearable research studies involving populations with dementia.

Multimedia Appendix 3 Presence and frequency of factors across wearable research studies involving populations with dementia.

## Abbreviations

MeSH: Medical Subject Headings
PICO: population, intervention, comparison, and outcomes
PRISMA: Preferred Reporting Items for Systematic Reviews and Meta-Analyses
QUEST: Quebec User Evaluation of Satisfaction With Assistive Technology
