# Supplementary material for: Enhancing Enrollment and Adherence in Long-Term Wearable Research on Dementia: Qualitative Systematic Review and Meta-Synthesis
Source: JMIR Aging. 2025 Jul 31;8:e63768. doi: 10.2196/63768 (PMC12355143; doi:10.2196/63768)
Supplement: Multimedia Appendix 2 [file aging_v8i1e63768_app2.docx]

*Multimedia Appendix 2. Summary of included wearable research studies involving populations with dementia*

| Study | Country | Study Type | Setting | Study Population | Device (Location) | Duration wear duration |
| --- | --- | --- | --- | --- | --- | --- |
| Ahmed et al. (2020) | Iraq | Observational | Community | unspecified group of "elder people" | proprietary device in development (not specified) | unknown |
| Amato et al. (2018) | Italy | Observational/qualitative | Alzheimer's village | 5 PwD | E4 bracelet by Empatica (wrist) | 10 weeks |
| Anderson et al. (2021) | USA | Observational | Community | 10 dyads | proprietary smartwatch part of BESI system (wrist) | 30 or 60 days |
| Berridge et al. (2022) | USA | Survey/qualitative | Community | 201 older adults "concerned about their own memory loss" from n=825 online cohort | n/a, survey | n/a |
| Bowen et al. (2021) | USA | Observational | Community | 17 older adults with MCI | GT3X+ by Actigraph (wrist) | 6 months |
| Buckley et al. (2020) | UK | Observational | Residential care | 257 PwD (higher level care, dementia care, or risk of wandering due to memory) | AX3 by Axivity (lower back at the fifth lumbar vertebrae adhered with hydrogel adhesive) | up to 7 days |
| Chen et al. (2019) | USA | Observational | Community | 897 older adults categorized as either cognitively normal, MCI, or dementia by MoCA from National Social Life, Health and Aging Project second wave cohort | unnamed actigraph (wrist) | 72 hours |
| Cohen et al. (2018) | USA | Observational | Community | 17 persons with Huntington’s Disease (one of two studies reported) | Apple Watch from Apple (wrist) | 6 months |
| Cruz-Sandoval et al. (2021) | USA | Observational/qualitative | Residential care | 10 PwDs 27 CGs, 1 CG supervisor | Fitbit: Charge 2 and Alta from Fitbit, Inc. (waist) | up to 71 days |
| Dai et al. (2019) | countries in Sub-Saharan Africa | Survey | not specified | 350 caregivers | n/a, survey | n/a |
| De Vito et al. (2020) | USA | Observational/qualitative | Residential care | 18 PwD; 6 paid caregivers | Fitbit Charge 2 HR from Fitbit, Inc. (waist) | 6 months |
| Dillon et al. (2021) | Canada | Trial | Assisted living | 25 PwD | 920 Actical accelerometer by Philips Respironics (not specified) | 10 weeks |
| Dinesh et al. (2020) | Canada | Observational | Community | 20 persons with Huntington's Disease, 19 controls | BioStampRC® wearable sensors from MC10 Inc (5, one on each limb and trunk/chest) | 2 days each after baseline, 6m, 9m, and 12m follow-ups |
| Eckert et al. (2020) | Germany | Trial | Community | 63 PwD | Digiwalker CW 700 pedometer by YAMAX (waist) | 12 weeks |
| Engelsma et al. (2022) | Netherlands | Qualitative | Community | 37 dementia case managers, informal caregivers, hospital healthcare professionals, district nurses, and researchers | n/a, Delphi study | n/a |
| Farina et al. (2019) | UK | Observational | Community | 26 PwD | GENEactiv Original from Activinsights Ltd. (wrist) | 1 month |
| Favela et al. (2020) | Mexico | Trial/qualitative | Residential care | 10 PwD; 9 paid caregivers | Fitbit Charge 2 and Alta form Fitbit Inc (waist) | 9 weeks |
| Freytag et al. (2022) | USA | Trial | Community | 19 PwD | PAMSys™ pendant from BioSensics, LLC (neck) | 48 hours |
| Gedde et al. (2021) | Norway | Trial | Community | 126 dyads | n/a, survey | n/a |
| Gelonch et al. (2019) | Spain | Observational/qualitative | Community | 9 dyads | Narrative Clip® lifelogging camera from Narrative Properties (neck) | 7 days |
| Gibson et al. (2019) | New Zealand | Observational | Community | 15 dyads | Actiwatch-2™ (non-dominant wrist) | 7 days |
| Godkin et al. (2022) | Canada | Observational/qualitative | Community | 13 PwD (out of 39 participants) | 4 limb wearables were GENEActiv Originals from ActivInsights; 1 chest Bittium Faros 180 from Bittium (2 wrists, 2 ankles and 1 chest device) fastened using rubber watch straps (wrists) or Fabrifoam medical-grade wraps (ankles); chest device was mounted using a 2-lead FastFix electrode adhesive | 7 days |
| Gris et al. (2023) | Italy | Qualitative | Community | 2 PwDs, 3 CGs, 7 healthcare professionals | n/a, focus groups | n/a |
| Guu, Aarseland et al. (2023) | UK | Observational/qualitative | Residential care | 28 PwD | GENEactiv Original from Activinsights Ltd. (wrist) | 4 weeks |
| Guu, Muurling, et al. (2023) | UK | Observational/qualitative | Community/residential care | STAND study n=29 PwD in residential care, RADAR-AD study n=175 PwD in the community | STAND = actigraphy watch (wrist); RADAR-AD two unnamed activity trackers | STAND = 8 weeks; RADAR-AD = 4 weeks |
| Hall et al. (2019) | UK | Qualitative | Residential care | 24 paid caregivers (clinicians, support workers), 9 family caregivers, 3 PwD | unnamed activity trackers | varied, 175 hours observed total |
| Hsu et al. (2023) | Taiwan | Observational/qualitative | Community | 5 different experiments 75-84 persons) | unspecified smart alert bracelet | 4 weeks |
| Jacklin et al. (2020) | Canada | Qualitative | Community | 29 (unspecified from Anishinaabe Elders, formal and informal caregivers, and health care providers) | CareBand from CareBand Inc (wrist) | n/a |
| Kaenampornpan et al. (2020) | Thailand | Qualitative | Community | unspecified doctors, caregivers | proprietary device in development (upper middle back) | n/a |
| Larnyo et al. (2022) | Ghana | Survey | Community | 262 PwD, 58 caregivers | n/a, survey | n/a |
| Lazarou et al. (2019) | Greece | Trial/qualitative | Community | 12 MCI, 6 PwD | Jawbone UP24 from Jawbone (wrist) | whole system installed 4-12 months |
| Lee et al. (2022) | USA | Observational | Community | 5 PwD | PAMSys+ pendant from BioSensics, LLC (neck) | 7 days |
| Mc Ardle et al. (2021) | UK | Trial | Community | 60 PwD; 25 controls | unspecified tri-axial accelerometer (lower back) | 7 days |
| Mc Ardle et al. (2018) | UK | Observational | Community | 20 PwD | AX3 from Axivity (lower back above fifth lumbar vertebra (L5) with double-sided tape and secured with Hypafix tape) | 7 days |
| McCarron et al. (2019) | USA | Trial/qualitative | Community | 48 PwD | unspecified smartwatch (wrist) | 6 months |
| Megges et al. (2018) | Germany | Observational | Community | 20 dyads | two GPS tracking watches: HIMATIC GPS Uhr Alpha and ReSOS-2—Die Notfalluhr (emergency watch) (wrist) | 4 weeks |
| Mishra et al. (2023) | USA | Survey/qualitative | Residential care or assisted living | 10 PwD, 14 caregivers, 11 dementia experts (e.g., geriatricians, social workers, psychologists, neurologists) | Care4AD pendant (neck) | n/a |
| Musaeus et al. (2022) | Denmark | Observational/qualitative | Community | 10 PwD | proprietary ear-EEG (six dry-electrodes mounted into custom 3D printed ear imprints fit to each participant) | 48 hours three times |
| Neubauer et al. (2022) | Canada | Qualitative | Community | 5 PwD, 5 caregivers, 6 dementia service providers, 5 technology developers | n/a, focus groups | n/a |
| Neubauer et al. (2021) | Canada | Qualitative | Community | 6 PwD, 6 caregivers, 6 paid caregivers, 4 social workers, 5 law enforcement and search and rescue personnel, 4 community organizers, 1 occupational therapist | n/a, focus groups | n/a |
| Nickerson et al. (2021) | USA | Trial | Community | 14 PwD | Activité from Withings | 12 weeks |
| O'Sullivan et al. (2023) | Ireland | Observational/qualitative | Community | 9 dyads | Fitbit Charge 3 from Fitbit Inc (waist) | 9 weeks |
| Parry et al. (2019) | Australia | Observational | Residential care | 8 PwD; 29 non-impaired residents | GT3X+ by Actigraph (right hip) | up to 7 days |
| Peeters et al. (2021) | Netherlands | Qualitative | Residential care | Stage 1: 8 formal caregivers (nurses) , 8 caregivers; Stage 2: 6 formal caregivers, 11 caregivers, 7 PwD , 2 case managers | E4 bracelet by Empatica (wrist) | 3-4 hours a day for 3 days |
| Raepsaet et al. (2021) | Belgium | Qualitative | Residential care | 10 caregivers (nurses) | hypothetical integrating sensors into disposable body-worn incontinence materials | n/a |
| Richeson et al. (2018) | USA | Observational/qualitative | Community | 10 MCI | FitBit Zip™ from Fitbit, Inc. (waist); Accusplit Eagle AC 120 XL Pedometer (waist) | 2 weeks |
| Rose et al. (2018) | USA | Qualitative | Community | 12 dyads | TEMPO (technology-enabled medical precision observation accelerometer) (wrist), DryBuddy incontinence sensor (underwear) | 5 to 7 nights |
| Sharma et al. (2023) | Netherlands | Observational/qualitative | Community | 464 PwD, 10 caregivers | n/a, survey and interviews | n/a |
| Snyder et al. (2020) | USA | Qualitative | Community | 7 caregivers | n/a, survey and interviews | n/a |
| Stavropoulos et al. (2021) | Multiple European countries | Qualitative | Community | 11 PwD, 10 caregivers | Four unspecified devices but pictured (wrist; but n/a, focus groups) | n/a |
| Stavropoulos et al. (2020) | Greece | Survey/qualitative | Community | 15 MCI, 15 caregivers, 15 health care providers | n/a, survey | n/a |
| Sun et al. (2021) | USA | Survey | Community | 202 caregivers | n/a, survey | n/a |
| Svetnik et al. (2021) | USA | Trial | Community | 285 PwD (probable) | Vívosmart® HR by Garmin (wrist) | 4 weeks |
| Thorpe et al. (2019) | Denmark | Observational/qualitative | Community | 6 PwD | SmartWatch 3 by Sony (wrist) | 8 weeks |
| Tiersen et al. (2021) | UK | Qualitative | Community | multiple sub-studies: 1) 9 PwD, 9 caregivers, 10 academic and clinical staff, 2) 35 dyads, 12 health and social workers 3) 14 occupational therapists, 4 National Health Service pathway directors, 6 researchers, 4) 8 health care services managers, 5) 2 PwD, 10 caregivers | unnamed smartwatch as part of system | n/a |
| van der Wardt et al. (2021) | UK | Trial/qualitative | Community | 60 PwD | Misfit Shine accelerometer from Misfit Wearables (wrist) | 7 days |
| Wangmo et al. (2019) | Switzerland, Germany, Italy | Qualitative | Community | 20 health care personnel and researchers (medical doctors, nurses and nursing home managers and researchers in the fields of geriatrics, psychiatry, neurology, neuropsychology, gerontology and nursing) | n/a, interviews | n/a |
| Wherton et al. (2019) | UK | Observational/qualitative | Community/residential care | 7 PwD | various: Buddi™ , Buddi Clip™, Vega GPS Watch™, Oysta Pearl+ Mobile™, Mindme™, GPS SmartSole™ (varied: wrist, lanyard, shoe) | 6 to 8 months |

Note: Dyad= a pair consisting of a caregiver and person living with dementia; PwD= person living with a form of dementia; MCI = Mild cognitive impairment

**Reference List with In-Text Reference Number**

46. Ahmed QA, Al-Neami AQH. A smart biomedical assisted system for Alzheimer patients. In: IOP Conference Series: Materials Science and Engineering. 2020:10.1088/1757-899X/881/1/012110.

33. Amato F, Crovari P, Masciadri A, et al. Clone: A promising system for the remote monitoring of Alzheimer’s patients an experimentation with a wearable device in a village for Alzheimer’s care. In: ACM International Conference Proceeding Series. 2018:255-260.doi:10.1145/3284869.3284906.

42. Anderson MS, Bankole A, Homdee N, Mitchell BA, Byfield GE, Lach J. Dementia caregiver experiences and recommendations for using the behavioral and environmental sensing and intervention system at home: Usability and acceptability study. JMIR Aging. 2021;4(4):e30353. doi:10.2196/30353. PMCID: PMC8691404.

63. Berridge C, Zhou Y, Lazar A, et al. Control matters in elder care technology: Evidence and direction for designing it in. Conference Paper. DIS (Des Interact Syst Conf). 2022:1831-1848. doi:10.1145/3532106.3533471. PMCID: PMC9367632.

74. Bowen ME, Gaynor B, Phillips LJ. Changes in physical and cognitive function predict sedentary behavior in older adults with mild cognitive impairment. Res Gerontol Nurs. 2021;14(6):285-291. doi:10.3928/19404921-20211021-01. PMCID: 34807787.

22. Buckley C, Cavadino A, Del Din S, et al. Quantifying reliable walking activity with a wearable device in aged residential care: How many days are enough? Sensors (Switzerland). 2020;20(21):1-12. 6314. doi:10.3390/s20216314. PMCID: 33167527.

70. Chen JH, Lauderdale DS. Cognitive function, consent for participation, and compliance with wearable device protocols in older adults. J Gerontol A Biol Sci Med Sci. 2019;74(2):269-273. doi:10.1093/gerona/gly032. PMCID: PMC6333929.

68. Cohen S, Waks Z, Elm JJ, et al. Characterizing patient compliance over six months in remote digital trials of Parkinson's and Huntington disease. BMC Med Inform Decis Mak. 2018;18(1):138. doi:10.1186/s12911-018-0714-7. PMCID: PMC6302308.

34. Cruz-Sandoval D, Favela J, Lopez-Nava IH, Morales A. Adoption of wearable devices by persons with dementia: Lessons from a non-pharmacological intervention enabled by a social robot. In: Marques G, Bhoi, A.K., Albuquerque, V.H.C.d., K.S., H. , ed. IOT in healthcare and ambient assisted living studies in computational intelligence. Springer; 2021:145-163.

43. Dai B, Larnyo E, Tetteh EA, Aboagye AK, Musah AA. Factors affecting caregivers' acceptance of the use of wearable devices by patients with dementia: An extension of the Unified Theory of Acceptance and Use of Technology model. Am J Alzheimers Dis Other Demen. 2020;35:1533317519883493. doi:10.1177/1533317519883493. PMCID: PMC10623900.

37. De Vito AN, Sawyer RJ, 2nd, LaRoche A, Arredondo B, Mizuki B, Knoop C. Acceptability and feasibility of a multicomponent telehealth care management program in older adults with advanced dementia in a residential memory care unit. Gerontol Geriatr Med. 2020;6:2333721420924988. doi:10.1177/2333721420924988. PMCID: PMC7288813.

71. Dillon K, Prapavessis H. Reducing sedentary behavior among mild to moderate cognitively impaired assisted living residents: A pilot randomized controlled trial (resedent study). J Aging Phys Act. 2021;29(1):27-35. doi:10.1123/japa.2019-0440. PMCID: 32580164.

60. Dinesh K, Snyder CW, Xiong M, et al. A longitudinal wearable sensor study in Huntington's disease. J Huntingtons Dis. 2020;9(1):69-81. doi:10.3233/jhd-190375. PMCID: 31868675.

69. Eckert T, Bongartz M, Ullrich P, et al. Promoting physical activity in geriatric patients with cognitive impairment after discharge from ward-rehabilitation: A feasibility study. Eur J Ageing. 2020;17(3):309-320. doi:10.1007/s10433-020-00555-w. PMCID: PMC7458987.

35. Engelsma T, Yurt A, Dröes RM, Jaspers MWM, Peute LW. Expert appraisal and prioritization of barriers to mhealth use for older adults living with Alzheimer’s disease and related dementias: A delphi study. Int J Med Inform. 2022;166:104845. doi:10.1016/j.ijmedinf.2022.104845. PMCID: 35973365.

47. Farina N, Sherlock G, Thomas S, Lowry RG, Banerjee S. Acceptability and feasibility of wearing activity monitors in community-dwelling older adults with dementia. Int J Geriatr Psychiatry. 2019;34(4):617-624. doi:10.1002/gps.5064. PMCID: 30701592.

20. Favela J, Cruz-Sandoval D, Morales-Tellez A, Lopez-Nava IH. Monitoring behavioral symptoms of dementia using activity trackers. J Biomed Inform. 2020;109103520. doi:10.1016/j.jbi.2020.103520. PMCID: 32783922.

52. Freytag J, Mishra RK, Street RL, Jr., et al. Using wearable sensors to measure goal achievement in older veterans with dementia. Sensors (Basel). 2022;22(24)doi:10.3390/s22249923. PMCID: PMC9782012.

75. Gedde MH, Husebo BS, Erdal A, et al. Access to and interest in assistive technology for home-dwelling people with dementia during the COVID-19 pandemic (pan.Dem). Int Rev Psychiatry. 2021;33(4):404-411. doi:10.1080/09540261.2020.1845620. PMCID: 33416012.

38. Gelonch O, Ribera M, Codern-Bove N, et al. Acceptability of a lifelogging wearable camera in older adults with mild cognitive impairment: A mixed-method study. BMC Geriatr. 2019;19(1):110. doi:10.1186/s12877-019-1132-0. PMCID: PMC6469032.

72. Gibson RH, Gander PH. Monitoring the sleep patterns of people with dementia and their family carers in the community. Australas J Ageing. Mar 2019;38(1):47-51. doi:10.1111/ajag.12605. PMCID: 30525266.

39. Godkin FE, Turner E, Demnati Y, et al. Feasibility of a continuous, multi-sensor remote health monitoring approach in persons living with neurodegenerative disease. J Neurol. 2022;269(5):2673-2686. doi:10.1007/s00415-021-10831-z. PMCID: PMC8548705.

44. Gris F, D'Amen B, Lamura G, Paciaroni L, Socci M, Melchiorre MG. Personalized technological support for informal caregivers of older people with dementia: A co-design approach involving potential end users and healthcare professionals in three focus groups in italy. Healthcare (Basel). 2023;11(19)doi:10.3390/healthcare11192640. PMCID: PMC10572801.

57. Guu T-W, Aarsland D, Ffytche DH. Feasibility of using wearable actigraphy for behavioural monitoring in care home residents living with moderate to severe Alzheimer’s disease and agitation. Alzheimers Dement. 2023;19(S19):e072283. doi:10.1002/Alz.072283.

9. Guu TW, Muurling M, Khan Z, et al. Wearable devices: Underrepresentation in the ageing society. Lancet Digit Health. 2023;5(6):e336-e337. doi:10.1016/s2589-7500(23)00069-9. PMCID: 37236695.

55. Hall A, Brown Wilson C, Stanmore E, Todd C. Moving beyond 'safety' versus 'autonomy': A qualitative exploration of the ethics of using monitoring technologies in long-term dementia care. BMC Geriatr. 2019;19(1):1-13. doi:10.1186/s12877-019-1155-6. PMCID: PMC6534927.

40. Hsu CK, Liu CC, Chang T, Liao JJ, Shu CM. Service design of a loss prevention device for older adults with dementia. Geriatrics (Basel). 2023;8(5):93. doi:10.3390/geriatrics8050093. PMCID: PMC10514846.

36. Jacklin K, Pitawanakwat K, Blind M, Lemieux AM, Sobol A, Warry W. Peace of mind: A community-industry-academic partnership to adapt dementia technology for Anishinaabe communities on Manitoulin Island. J Rehabil Assist Technol Eng. 2020;7:2055668320958327. doi:10.1177/2055668320958327. PMCID: PMC7509219.

77. Kaenampornpan M, Khai ND, Kawattikul K. Wearable computing for dementia patients. Conference Paper 2020; 10.1007/978-3-030-44044-2_3.

45. Larnyo E, Dai B, Larnyo A, et al. Impact of actual use behavior of healthcare wearable devices on quality of life: A cross-sectional survey of people with dementia and their caregivers in ghana. Healthcare (Basel). 2022;10(2)doi:10.3390/healthcare10020275. PMCID: PMC8872618.

25. Lazarou I, Stavropoulos TG, Meditskos G, Andreadis S, Kompatsiaris IY, Tsolaki M. Long-term impact of intelligent monitoring technology on people with cognitive impairment: An observational study. J Alzheimers Dis. 2019;70(3):757-792. doi:10.3233/JAD-190423. PMCID: 31256141.

26. Lee M, Mishra RK, Momin A, et al. Smart-home concept for remote monitoring of instrumental activities of daily living (IADL) in older adults with cognitive impairment: A proof of concept and feasibility study. Sensors. 2022;22(18)6745. doi:10.3390/s22186745. PMCID: PMC9501541.

54. Mc Ardle R, Del Din S, Donaghy P, Galna B, Thomas AJ, Rochester L. The impact of environment on gait assessment: Considerations from real-world gait analysis in dementia subtypes. Sensors (Switzerland). 2021;21(3):1-15. 813. doi:10.3390/s21030813. PMCID: PMC7865394.

61. Mc Ardle R, Morris R, Hickey A, et al. Gait in mild Alzheimer’s disease: Feasibility of multi-center measurement in the clinic and home with body-worn sensors: A pilot study. J Alzheimers Dis. 2018;63(1):331-341. doi:10.3233/JAD-171116. PMCID: PMC7617011.

51. McCarron HR, Zmora R, Gaugler JE. A web-based mobile app with a smartwatch to support social engagement in persons with memory loss: Pilot randomized controlled trial. JMIR Aging. 2019;2(1):e13378. doi:10.2196/13378. PMCID: PMC6715400.

48. Megges H, Freiesleben SD, Rösch C, Knoll N, Wessel L, Peters O. User experience and clinical effectiveness with two wearable global positioning system devices in home dementia care. Alzheimers Dement (N Y). 2018;4:636-644. doi:10.1016/j.trci.2018.10.002. PMCID: PMC6260223.

27. Mishra RK, Park C, Momin AS, et al. Care4AD: A technology-driven platform for care coordination and management: Acceptability study in dementia. Gerontology. 2023;69(2):227-238. doi:10.1159/000526219. PMCID: 36096091.

59. Musaeus CS, Waldemar G, Andersen BB, et al. Long-term EEG monitoring in patients with Alzheimer’s disease using ear-EEG: A feasibility study. J Alzheimers Dis. 2022;90(4):1713-1723. doi:10.3233/JAD-220491. PMCID: 36336927.

10. Neubauer N, Spenrath C, Philip S, Daum C, Liu L, Miguel-Cruz A. Identifying adoption and usability factors of locator devices for persons living with dementia. Dementia (London). 2022;21(3):862-881. doi:10.1177/14713012211065381. PMCID: PMC8996292.

18. Neubauer NA, Liu L. Influence of perspectives on user adoption of wander-management strategies. Dementia (London). 2021;20(2):734-758. doi:10.1177/1471301220911304. PMCID: 32164446.

67. Nickerson GY, Shade K. Effect of increasing physical activity on cognitive function in individuals with mild cognitive impairment: A knowledge translation to practice pilot project. J Gerontol Nurs. 2021;47(5):14-18. doi:10.3928/00989134-20210401-01. PMCID: 34039095.
