# Supplementary material for: Enhancing Enrollment and Adherence in Long-Term Wearable Research on Dementia: Qualitative Systematic Review and Meta-Synthesis
Source: JMIR Aging. 2025 Jul 31;8:e63768. doi: 10.2196/63768 (PMC12355143; doi:10.2196/63768)
Supplement: Multimedia Appendix 3 [file aging_v8i1e63768_app3.docx]

*Multimedia Appendix 3. Presence and frequency of factors across wearable research studies involving populations with dementia*

|  | Device selection | | | | | | | | | | Protocol considerations | | | | | | Enhancing recruitment | | | | | | Promoting adherence | | | | | | |  | | |  |
| --- | --- | --- | --- | --- | --- | --- | --- | --- | --- | --- | --- | --- | --- | --- | --- | --- | --- | --- | --- | --- | --- | --- | --- | --- | --- | --- | --- | --- | --- | --- | --- | --- | --- |
| Study | **Easy to use** | **Smaller size/weight** | **Comfort** | **Fits into routine** | **Unobtrusive** | **Tailorable** | **Materials** | **Aesthetics** | **Easy to wear** | **Water resistant** | **Privacy concerns** | **Consent capacity** | **Battery** | **Adjustment period** | **Task requirements** | **Technical anxiety** | **Multifunctional** | **Burden impact** | **Stigma** | **Caregivers buy-in** | **Connectivity** | **Remote monitoring** | **Provide health insight** | **Technical support** | **Caregivers support** | **Safety** | **Remembering the device** | **Independence** | **Self-removal** | Total | |  |  |
| Ahmed et al. (2020) |  | **•** |  |  |  |  |  |  |  |  |  |  |  |  |  |  |  |  |  |  |  | **•** |  |  |  |  |  |  |  | 2 | |  |  |
| Amato et al. (2018) | **•** |  | **•** |  |  |  | **•** |  |  | **•** |  |  | **•** |  |  |  |  |  |  |  |  |  |  |  |  | **•** |  |  |  | 6 | |  |  |
| Anderson et al. (2021) | **•** |  |  |  |  |  |  |  |  |  |  |  |  |  |  | **•** |  |  |  | **•** |  |  |  |  | **•** |  |  |  |  | 4 | |  |  |
| Berridge et al. (2022) |  |  |  |  |  |  |  |  |  |  | **•** | **•** |  |  |  |  |  |  |  |  |  |  |  |  |  |  |  |  |  | 2 | |  |  |
| Bowen et al. (2021) |  |  |  |  |  |  |  |  |  |  |  |  |  |  |  |  |  | **•** |  |  |  |  | **•** |  |  | **•** | **•** |  |  | 4 | |  |  |
| Buckley et al. (2020) |  |  |  |  |  |  | **•** |  |  |  |  | **•** |  |  |  |  |  |  |  |  |  |  |  |  |  |  | **•** |  | **•** | 4 | |  |  |
| Chen et al. (2019) |  |  |  |  |  |  |  |  |  |  |  |  |  |  | **•** |  |  |  |  |  |  |  |  |  |  |  |  |  |  | 1 | |  |  |
| Cohen et al. (2018) |  |  |  |  |  |  |  |  |  |  |  |  |  | **•** |  | **•** |  |  |  |  |  |  | **•** | **•** |  |  | **•** |  |  | 5 | |  |  |
| Cruz-Sandoval et al. (2021) | **•** |  | **•** | **•** | **•** | **•** | **•** |  |  | **•** |  |  | **•** | **•** |  | **•** | **•** | **•** | **•** | **•** |  |  | **•** | **•** | **•** |  | **•** | **•** |  | 19 | |  |  |
| Dai et al. (2019) | **•** |  |  |  |  |  |  |  |  |  |  |  |  |  |  | **•** |  |  | **•** |  |  |  |  | **•** |  |  |  |  |  | 4 | |  |  |
| De Vito et al. (2020) | **•** |  | **•** | **•** |  |  |  |  |  |  |  | **•** | **•** | **•** |  |  | **•** | **•** |  | **•** |  | **•** | **•** |  |  |  |  |  | **•** | 12 | |  |  |
| Dillon et al. (2021) |  |  |  |  |  |  |  |  |  |  |  |  |  |  | **•** |  |  |  |  |  | **•** |  |  |  |  |  |  |  |  | 2 | |  |  |
| Dinesh et al. (2020) |  |  |  |  |  |  |  |  | **•** |  |  |  |  |  | **•** |  |  |  |  |  |  |  |  |  |  |  |  |  |  | 2 | |  |  |
| Eckert et al. (2020) |  |  |  |  |  |  |  |  |  |  |  |  |  | **•** |  |  |  |  |  |  |  |  | **•** |  |  |  |  |  |  | 2 | |  |  |
| Engelsma et al. (2022) | **•** |  |  | **•** |  |  |  |  |  |  |  | **•** |  |  |  | **•** | **•** |  | **•** |  |  |  |  |  |  |  |  |  |  | 6 | |  |  |
| Farina et al. (2019) |  | **•** | **•** | **•** |  |  | **•** | **•** |  |  |  |  |  | **•** |  |  | **•** |  |  | **•** |  |  |  |  | **•** |  |  |  |  | 9 | |  |  |
| Favela et al. (2020) |  |  | **•** | **•** | **•** |  |  |  |  |  |  | **•** |  |  |  | **•** |  |  |  | **•** |  |  |  | **•** | **•** |  | **•** |  |  | 9 | |  |  |
| Freytag et al. (2022) |  |  | **•** |  |  |  |  |  |  | **•** |  |  |  |  |  |  |  |  |  |  |  |  |  |  | **•** |  |  |  | **•** | 4 | |  |  |
| Gedde et al. (2021) |  |  |  |  |  |  |  |  |  |  |  |  |  |  |  |  |  | **•** |  |  |  | **•** |  |  |  | **•** |  |  |  | 3 | |  |  |
| Gelonch et al. (2019) | **•** |  | **•** | **•** |  |  |  |  |  |  | **•** |  |  | **•** |  |  |  | **•** | **•** |  |  |  | **•** | **•** | **•** |  |  | **•** |  | 11 | |  |  |
| Gibson et al. (2019) |  |  |  |  |  |  |  |  |  |  |  |  |  |  | **•** |  |  |  |  | **•** |  |  |  |  | **•** |  |  |  |  | 3 | |  |  |
| Godkin et al. (2022) | **•** | **•** | **•** |  |  | **•** | **•** | **•** | **•** | **•** |  |  |  |  |  |  | **•** |  | **•** |  |  |  | **•** |  | **•** | **•** | **•** |  |  | 14 | |  |  |
| Gris et al. (2023) | **•** | **•** | **•** | **•** | **•** | **•** |  |  |  |  | **•** | **•** | **•** |  |  | **•** |  |  |  |  |  | **•** | **•** | **•** |  |  |  | **•** |  | 14 | |  |  |
| Guu, Aarseland et al. (2023) |  |  |  | **•** |  |  |  | **•** |  |  |  |  |  |  |  |  |  |  |  | **•** |  |  |  | **•** |  |  |  |  |  | 4 | |  |  |
| Guu, Muurling, et al. (2023) |  |  |  |  |  |  |  |  |  |  |  |  | **•** | **•** | **•** |  |  |  |  |  |  |  |  | **•** |  |  |  |  |  | 4 | |  |  |
| Hall et al. (2019) |  |  |  | **•** |  |  |  |  |  |  |  |  |  |  |  | **•** |  |  |  |  |  | **•** |  |  |  | **•** |  |  |  | 4 | |  |  |
| Hsu et al. (2023) | **•** | **•** |  | **•** | **•** | **•** |  | **•** |  |  |  |  | **•** |  |  |  | **•** |  |  |  |  |  |  |  | **•** |  |  |  |  | 9 | |  |  |
| Jacklin et al. (2020) | **•** | **•** | **•** |  |  | **•** |  | **•** |  |  | **•** |  |  |  |  |  | **•** |  |  |  | **•** | **•** |  |  |  | **•** |  |  |  | 10 | |  |  |
| Kaenampornpan et al. (2020) |  |  |  |  |  |  |  |  |  |  |  |  |  |  |  |  |  |  |  |  |  |  |  |  |  |  |  |  | **•** | • | |  |  |
| Larnyo et al. (2022) | **•** |  |  |  |  |  |  |  |  |  |  |  |  |  |  |  |  | **•** | **•** | **•** |  |  |  | **•** | **•** |  |  | **•** |  | 7 | |  |  |
| Lazarou et al. (2019) | **•** |  |  |  | **•** |  |  |  |  |  | **•** |  |  |  |  |  | **•** |  |  |  |  | **•** | **•** |  |  |  |  |  |  | 6 | |  |  |
| Lee et al. (2022) | **•** |  |  |  |  | **•** |  |  |  |  | **•** |  |  |  |  |  |  |  |  |  |  |  |  | **•** |  |  |  |  |  | 4 | |  |  |
| Mc Ardle et al. (2021) |  |  | **•** |  |  |  |  |  |  |  |  |  |  |  |  |  |  |  |  |  |  |  |  | **•** |  |  |  |  |  | 2 | |  |  |
| Mc Ardle et al. (2018) |  |  |  |  |  |  |  |  | **•** |  |  |  |  |  | **•** |  |  |  |  |  |  |  |  | **•** |  |  |  |  |  | 3 | |  |  |
| McCarron et al. (2019) |  | **•** |  |  |  |  |  | **•** |  |  |  | **•** |  |  |  |  |  |  |  |  |  |  |  |  |  |  | **•** |  |  | 4 | |  |  |
| Megges et al. (2018) |  | **•** |  |  | **•** |  |  |  |  |  |  |  |  | **•** |  | **•** |  |  | **•** |  |  | **•** |  |  |  |  |  |  |  | 6 | |  |  |
| Mishra et al. (2023) | **•** | **•** |  |  |  |  |  |  |  |  | **•** |  |  |  |  | **•** | **•** | **•** |  |  |  | **•** | **•** |  |  |  |  | **•** |  | 8 | |  |  |
| Musaeus et al. (2022) |  | **•** |  |  |  |  | **•** |  | **•** |  |  |  |  |  |  |  |  |  |  |  |  |  |  |  | **•** | **•** |  |  |  | 5 | |  |  |
| Neubauer et al. (2022) | **•** | **•** |  | **•** | **•** | **•** | **•** |  | **•** |  | **•** | **•** | **•** |  |  | **•** | **•** |  | **•** |  | **•** | **•** | **•** | **•** |  |  | **•** | **•** |  | 5 | |  |  |
| Neubauer et al. (2021) |  |  |  |  |  | **•** |  |  |  |  | **•** |  |  |  |  | **•** |  |  | **•** |  | **•** |  |  |  |  |  |  |  |  | 19 | |  |  |
| Nickerson et al. (2021) |  |  |  |  |  |  |  |  |  |  |  |  | **•** |  |  |  | **•** |  |  |  |  |  |  |  |  |  |  |  |  | 2 | |  |  |
|  | **Device selection** | | | | | | | | | | **Protocol considerations** | | | | | | **Enhancing recruitment** | | | | | | **Promoting adherence** | | | | | | |  | | |  |
| Study | **Easy to use** | **Smaller size/weight** | **Comfort** | **Fits into routine** | **Unobtrusive** | **Tailorable** | **Materials** | **Aesthetics** | **Easy to wear** | **Water resistant** | **Privacy concerns** | **Consent capacity** | **Battery** | **Adjustment period** | **Task requirements** | **Technical anxiety** | **Multifunctional** | **Burden impact** | **Stigma** | **Caregivers buy-in** | **Connectivity** | **Remote monitoring** | **Provide health insight** | **Technical support** | **Caregivers support** | **Safety** | **Remembering the device** | **Independence** | **Self-removal** | Total | |  |  |
| O'Sullivan et al. (2023) |  |  |  |  |  |  |  |  |  |  |  |  |  |  |  |  |  |  |  |  | **•** | **•** | **•** | **•** | **•** |  | **•** |  |  | 6 | |  |  |
| Parry et al. (2019) |  |  |  | **•** | **•** |  |  |  |  |  |  | **•** |  |  |  |  |  | **•** |  | **•** |  |  |  |  |  |  |  |  |  | 5 | |  |  |
| Peeters et al. (2021) | **•** | **•** | **•** |  | **•** | **•** | **•** |  |  | **•** | **•** |  |  | **•** |  |  |  | **•** | **•** | **•** | **•** | **•** | **•** |  |  |  |  |  | **•** | 16 | |  |  |
| Raepsaet et al. (2021) |  | **•** | **•** |  |  |  |  |  |  |  |  | **•** |  |  |  |  |  | **•** |  |  | **•** | **•** | **•** |  |  | **•** |  |  |  | 8 | |  |  |
| Richeson et al. (2018) | **•** |  |  |  |  | **•** |  |  | **•** |  |  |  |  |  |  | **•** |  |  |  |  |  |  | **•** |  |  |  |  |  |  | 5 | |  |  |
| Rose et al. (2018) | **•** | **•** | **•** |  | **•** |  | **•** |  |  |  |  |  |  |  |  |  |  |  |  |  |  |  |  | **•** |  |  |  |  | **•** | 7 | |  |  |
| Sharma et al. (2023) |  |  |  |  |  |  |  |  |  |  | **•** |  | **•** |  | **•** |  |  | **•** |  | **•** |  | **•** |  |  |  | **•** |  |  | **•** | 8 | |  |  |
| Snyder et al. (2020) |  |  |  |  | **•** | **•** |  |  |  |  | **•** | **•** |  |  |  | **•** | **•** |  |  |  |  |  |  |  | **•** | **•** |  | **•** |  | 9 | |  |  |
| Stavropoulos et al. (2021) |  | **•** |  |  |  |  | **•** | **•** |  | **•** | **•** |  | **•** |  |  | **•** | **•** | **•** |  |  |  | **•** | **•** |  |  | **•** | **•** | **•** |  | 14 | |  |  |
| Stavropoulos et al. (2020) | **•** | **•** |  |  |  |  |  | **•** |  | **•** |  |  | **•** |  |  |  | **•** | **•** | **•** |  |  | **•** | **•** |  |  | **•** |  | **•** |  | 12 | |  |  |
| Sun et al. (2021) |  |  |  |  |  |  |  |  |  |  |  |  |  |  |  | **•** |  |  | **•** | **•** |  | **•** |  |  |  | **•** |  |  |  | 5 | |  |  |
| Svetnik et al. (2021) |  |  |  |  |  |  |  |  |  |  |  |  | **•** |  | **•** |  |  |  |  |  | **•** |  |  |  |  |  | **•** |  |  | 4 | |  |  |
| Thorpe et al. (2019) |  | **•** | **•** | **•** |  |  |  |  |  |  |  |  |  |  |  |  | **•** | **•** |  |  | **•** | **•** |  |  |  | **•** |  | **•** |  | 9 | |  |  |
| Tiersen et al. (2021) |  |  |  |  |  |  |  |  |  |  |  | **•** |  |  | **•** | **•** | **•** | **•** |  |  | **•** | **•** | **•** | **•** |  |  |  |  |  | 9 | |  |  |
| van der Wardt et al. (2021) | **•** |  |  |  |  |  |  |  | **•** |  |  |  |  | **•** |  |  |  | **•** |  |  |  |  |  |  |  |  | **•** |  |  | 5 | |  |  |
| Wangmo et al. (2019) |  |  |  |  |  |  |  |  |  |  | **•** | **•** |  |  |  |  |  |  |  |  |  |  |  |  |  |  |  |  |  | 2 | |  |  |
| Wherton et al. (2019) |  | **•** |  |  | **•** | **•** |  |  |  |  | **•** | **•** | **•** |  |  |  |  |  | **•** |  |  |  |  | **•** | **•** |  |  |  |  | 9 | |  |  |
| Total | 21 | 18 | 15 | 13 | 12 | 12 | 10 | 8 | 7 | 7 | 15 | 14 | 13 | 10 | 9 | 17 | 16 | 16 | 13 | 12 | 10 | 19 | 18 | 17 | 14 | 14 | 12 | 10 | 7 |  |  | | |

**Reference List with In-Text Reference Number**

46. Ahmed QA, Al-Neami AQH. A smart biomedical assisted system for Alzheimer patients. In: IOP Conference Series: Materials Science and Engineering. 2020:10.1088/1757-899X/881/1/012110.

33. Amato F, Crovari P, Masciadri A, et al. Clone: A promising system for the remote monitoring of Alzheimer’s patients an experimentation with a wearable device in a village for Alzheimer’s care. In: ACM International Conference Proceeding Series. 2018:255-260.doi:10.1145/3284869.3284906.

42. Anderson MS, Bankole A, Homdee N, Mitchell BA, Byfield GE, Lach J. Dementia caregiver experiences and recommendations for using the behavioral and environmental sensing and intervention system at home: Usability and acceptability study. JMIR Aging. 2021;4(4):e30353. doi:10.2196/30353. PMCID: PMC8691404.

63. Berridge C, Zhou Y, Lazar A, et al. Control matters in elder care technology: Evidence and direction for designing it in. Conference Paper. DIS (Des Interact Syst Conf). 2022:1831-1848. doi:10.1145/3532106.3533471. PMCID: PMC9367632.

74. Bowen ME, Gaynor B, Phillips LJ. Changes in physical and cognitive function predict sedentary behavior in older adults with mild cognitive impairment. Res Gerontol Nurs. 2021;14(6):285-291. doi:10.3928/19404921-20211021-01. PMCID: 34807787.

22. Buckley C, Cavadino A, Del Din S, et al. Quantifying reliable walking activity with a wearable device in aged residential care: How many days are enough? Sensors (Switzerland). 2020;20(21):1-12. 6314. doi:10.3390/s20216314. PMCID: 33167527.

70. Chen JH, Lauderdale DS. Cognitive function, consent for participation, and compliance with wearable device protocols in older adults. J Gerontol A Biol Sci Med Sci. 2019;74(2):269-273. doi:10.1093/gerona/gly032. PMCID: PMC6333929.

68. Cohen S, Waks Z, Elm JJ, et al. Characterizing patient compliance over six months in remote digital trials of Parkinson's and Huntington disease. BMC Med Inform Decis Mak. 2018;18(1):138. doi:10.1186/s12911-018-0714-7. PMCID: PMC6302308.

34. Cruz-Sandoval D, Favela J, Lopez-Nava IH, Morales A. Adoption of wearable devices by persons with dementia: Lessons from a non-pharmacological intervention enabled by a social robot. In: Marques G, Bhoi, A.K., Albuquerque, V.H.C.d., K.S., H. , ed. IOT in healthcare and ambient assisted living studies in computational intelligence. Springer; 2021:145-163.

43. Dai B, Larnyo E, Tetteh EA, Aboagye AK, Musah AA. Factors affecting caregivers' acceptance of the use of wearable devices by patients with dementia: An extension of the Unified Theory of Acceptance and Use of Technology model. Am J Alzheimers Dis Other Demen. 2020;35:1533317519883493. doi:10.1177/1533317519883493. PMCID: PMC10623900.

37. De Vito AN, Sawyer RJ, 2nd, LaRoche A, Arredondo B, Mizuki B, Knoop C. Acceptability and feasibility of a multicomponent telehealth care management program in older adults with advanced dementia in a residential memory care unit. Gerontol Geriatr Med. 2020;6:2333721420924988. doi:10.1177/2333721420924988. PMCID: PMC7288813.

71. Dillon K, Prapavessis H. Reducing sedentary behavior among mild to moderate cognitively impaired assisted living residents: A pilot randomized controlled trial (resedent study). J Aging Phys Act. 2021;29(1):27-35. doi:10.1123/japa.2019-0440. PMCID: 32580164.

60. Dinesh K, Snyder CW, Xiong M, et al. A longitudinal wearable sensor study in Huntington's disease. J Huntingtons Dis. 2020;9(1):69-81. doi:10.3233/jhd-190375. PMCID: 31868675.

69. Eckert T, Bongartz M, Ullrich P, et al. Promoting physical activity in geriatric patients with cognitive impairment after discharge from ward-rehabilitation: A feasibility study. Eur J Ageing. 2020;17(3):309-320. doi:10.1007/s10433-020-00555-w. PMCID: PMC7458987.

35. Engelsma T, Yurt A, Dröes RM, Jaspers MWM, Peute LW. Expert appraisal and prioritization of barriers to mhealth use for older adults living with Alzheimer’s disease and related dementias: A delphi study. Int J Med Inform. 2022;166:104845. doi:10.1016/j.ijmedinf.2022.104845. PMCID: 35973365.

47. Farina N, Sherlock G, Thomas S, Lowry RG, Banerjee S. Acceptability and feasibility of wearing activity monitors in community-dwelling older adults with dementia. Int J Geriatr Psychiatry. 2019;34(4):617-624. doi:10.1002/gps.5064. PMCID: 30701592.

20. Favela J, Cruz-Sandoval D, Morales-Tellez A, Lopez-Nava IH. Monitoring behavioral symptoms of dementia using activity trackers. J Biomed Inform. 2020;109103520. doi:10.1016/j.jbi.2020.103520. PMCID: 32783922.

52. Freytag J, Mishra RK, Street RL, Jr., et al. Using wearable sensors to measure goal achievement in older veterans with dementia. Sensors (Basel). 2022;22(24)doi:10.3390/s22249923. PMCID: PMC9782012.

75. Gedde MH, Husebo BS, Erdal A, et al. Access to and interest in assistive technology for home-dwelling people with dementia during the COVID-19 pandemic (pan.Dem). Int Rev Psychiatry. 2021;33(4):404-411. doi:10.1080/09540261.2020.1845620. PMCID: 33416012.

38. Gelonch O, Ribera M, Codern-Bove N, et al. Acceptability of a lifelogging wearable camera in older adults with mild cognitive impairment: A mixed-method study. BMC Geriatr. 2019;19(1):110. doi:10.1186/s12877-019-1132-0. PMCID: PMC6469032.

72. Gibson RH, Gander PH. Monitoring the sleep patterns of people with dementia and their family carers in the community. Australas J Ageing. Mar 2019;38(1):47-51. doi:10.1111/ajag.12605. PMCID: 30525266.

39. Godkin FE, Turner E, Demnati Y, et al. Feasibility of a continuous, multi-sensor remote health monitoring approach in persons living with neurodegenerative disease. J Neurol. 2022;269(5):2673-2686. doi:10.1007/s00415-021-10831-z. PMCID: PMC8548705.

44. Gris F, D'Amen B, Lamura G, Paciaroni L, Socci M, Melchiorre MG. Personalized technological support for informal caregivers of older people with dementia: A co-design approach involving potential end users and healthcare professionals in three focus groups in italy. Healthcare (Basel). 2023;11(19)doi:10.3390/healthcare11192640. PMCID: PMC10572801.

57. Guu T-W, Aarsland D, Ffytche DH. Feasibility of using wearable actigraphy for behavioural monitoring in care home residents living with moderate to severe Alzheimer’s disease and agitation. Alzheimers Dement. 2023;19(S19):e072283. doi:10.1002/Alz.072283.

9. Guu TW, Muurling M, Khan Z, et al. Wearable devices: Underrepresentation in the ageing society. Lancet Digit Health. 2023;5(6):e336-e337. doi:10.1016/s2589-7500(23)00069-9. PMCID: 37236695.

55. Hall A, Brown Wilson C, Stanmore E, Todd C. Moving beyond 'safety' versus 'autonomy': A qualitative exploration of the ethics of using monitoring technologies in long-term dementia care. BMC Geriatr. 2019;19(1):1-13. doi:10.1186/s12877-019-1155-6. PMCID: PMC6534927.

40. Hsu CK, Liu CC, Chang T, Liao JJ, Shu CM. Service design of a loss prevention device for older adults with dementia. Geriatrics (Basel). 2023;8(5):93. doi:10.3390/geriatrics8050093. PMCID: PMC10514846.

36. Jacklin K, Pitawanakwat K, Blind M, Lemieux AM, Sobol A, Warry W. Peace of mind: A community-industry-academic partnership to adapt dementia technology for Anishinaabe communities on Manitoulin Island. J Rehabil Assist Technol Eng. 2020;7:2055668320958327. doi:10.1177/2055668320958327. PMCID: PMC7509219.

77. Kaenampornpan M, Khai ND, Kawattikul K. Wearable computing for dementia patients. Conference Paper 2020; 10.1007/978-3-030-44044-2_3.

45. Larnyo E, Dai B, Larnyo A, et al. Impact of actual use behavior of healthcare wearable devices on quality of life: A cross-sectional survey of people with dementia and their caregivers in ghana. Healthcare (Basel). 2022;10(2)doi:10.3390/healthcare10020275. PMCID: PMC8872618.

25. Lazarou I, Stavropoulos TG, Meditskos G, Andreadis S, Kompatsiaris IY, Tsolaki M. Long-term impact of intelligent monitoring technology on people with cognitive impairment: An observational study. J Alzheimers Dis. 2019;70(3):757-792. doi:10.3233/JAD-190423. PMCID: 31256141.

26. Lee M, Mishra RK, Momin A, et al. Smart-home concept for remote monitoring of instrumental activities of daily living (IADL) in older adults with cognitive impairment: A proof of concept and feasibility study. Sensors. 2022;22(18)6745. doi:10.3390/s22186745. PMCID: PMC9501541.

54. Mc Ardle R, Del Din S, Donaghy P, Galna B, Thomas AJ, Rochester L. The impact of environment on gait assessment: Considerations from real-world gait analysis in dementia subtypes. Sensors (Switzerland). 2021;21(3):1-15. 813. doi:10.3390/s21030813. PMCID: PMC7865394.

61. Mc Ardle R, Morris R, Hickey A, et al. Gait in mild Alzheimer’s disease: Feasibility of multi-center measurement in the clinic and home with body-worn sensors: A pilot study. J Alzheimers Dis. 2018;63(1):331-341. doi:10.3233/JAD-171116. PMCID: PMC7617011.

51. McCarron HR, Zmora R, Gaugler JE. A web-based mobile app with a smartwatch to support social engagement in persons with memory loss: Pilot randomized controlled trial. JMIR Aging. 2019;2(1):e13378. doi:10.2196/13378. PMCID: PMC6715400.

48. Megges H, Freiesleben SD, Rösch C, Knoll N, Wessel L, Peters O. User experience and clinical effectiveness with two wearable global positioning system devices in home dementia care. Alzheimers Dement (N Y). 2018;4:636-644. doi:10.1016/j.trci.2018.10.002. PMCID: PMC6260223.

27. Mishra RK, Park C, Momin AS, et al. Care4AD: A technology-driven platform for care coordination and management: Acceptability study in dementia. Gerontology. 2023;69(2):227-238. doi:10.1159/000526219. PMCID: 36096091.

59. Musaeus CS, Waldemar G, Andersen BB, et al. Long-term EEG monitoring in patients with Alzheimer’s disease using ear-EEG: A feasibility study. J Alzheimers Dis. 2022;90(4):1713-1723. doi:10.3233/JAD-220491. PMCID: 36336927.

10. Neubauer N, Spenrath C, Philip S, Daum C, Liu L, Miguel-Cruz A. Identifying adoption and usability factors of locator devices for persons living with dementia. Dementia (London). 2022;21(3):862-881. doi:10.1177/14713012211065381. PMCID: PMC8996292.

18. Neubauer NA, Liu L. Influence of perspectives on user adoption of wander-management strategies. Dementia (London). 2021;20(2):734-758. doi:10.1177/1471301220911304. PMCID: 32164446.

67. Nickerson GY, Shade K. Effect of increasing physical activity on cognitive function in individuals with mild cognitive impairment: A knowledge translation to practice pilot project. J Gerontol Nurs. 2021;47(5):14-18. doi:10.3928/00989134-20210401-01. PMCID: 34039095.

76. O'Sullivan G, Whelan B, Gallagher N, et al. Challenges of using a Fitbit smart wearable among people with dementia. Int J Geriatr Psychiatry. 2023;38(3):e5898. doi:10.1002/gps.5898. PMCID: 36814072.

56. Parry S, Chow M, Batchelor F, Fary RE. Physical activity and sedentary behaviour in a residential aged care facility. Australas J Ageing. 2019;38(1):E12-E18. doi:10.1111/ajag.12589. PMCID: 30281184.

28. Peeters MWH, Schouten G, Wouters EJ. Wearables for residents of nursing homes with dementia and challenging behaviour: Values, attitudes, and needs. Gerontechnology. 2021;20(2):1-13. doi:10.4017/GT.2021.20.2.7.06.

53. Raepsaet C, Serraes B, Verhaeghe S, Beeckman D. Integrating sensor technology in disposable body-worn absorbent products: A qualitative study to define user profile, (technical) criteria, conditions, and potential benefits. J Wound Ostomy Continence Nurs. 2021;48(6):560-567. doi:10.1097/won.0000000000000812. PMCID: 34781313.

29. Richeson NE, Croteau KA. A feasibility study examining use of the Fitbit Zip™ vs. The Accusplit Eagle AC 120 XL Pedometer to increase physical activity for persons with mild cognitive disorder. AAA. 2017;42(1):41-53. doi:10.1080/01924788.2017.1385367.

41. Rose KM, Lach J, Perkhounkova Y, et al. Use of body sensors to examine nocturnal agitation, sleep, and urinary incontinence in individuals with Alzheimer’s disease. J Gerontol Nurs. 2018;44(8):19-26. doi:10.3928/00989134-20180626-03. PMCID: 30059136.

62. Sharma N, Braakman-Jansen LMA, Oinas-Kukkonen H, Croockewit JH, Gemert-Pijnen JV. Exploring the needs and requirements of informal caregivers of older adults with cognitive impairment from sensor-based care solutions: Multimethod study. JMIR Aging. 2023;6:e49319. doi:10.2196/49319. PMCID: PMC10632915.

58. Snyder M, Dringus L, Schladen MM, Chenail R, Oviawe E. Remote monitoring technologies in dementia care: An interpretative phenomenological analysis of family caregivers’ experiences. Qual Rep. 2020;25(5):1233-1252. doi:10.46743/2160-3715/2020.4127.

30. Stavropoulos TG, Lazarou I, Diaz A, et al. Wearable devices for assessing function in Alzheimer's disease: A European public involvement activity about the features and preferences of patients and caregivers. Front Aging Neurosci. 2021;13:643135. doi:10.3389/fnagi.2021.643135. PMCID: PMC8072390.

31. Stavropoulos TG, Lazarou I, Strantsalis D, et al. Human factors and requirements of people with mild cognitive impairment, their caregivers and healthcare professionals for eHealth systems with wearable trackers. In: Proceedings of the 2020 IEEE International Conference on Human-Machine Systems, ICHMS 2020. 2020:10.1109/ICHMS49158.2020.9209340.

73. Sun Y, Wang Y, Kim HM, et al. GPS tracking in dementia caregiving: Social norm, perceived usefulness, and behavioral intent to use technology. In: Proceedings of the Annual Hawaii International Conference on System Sciences. 2021:3804-3817.doi:10.24251/HICSS.2021.461.

66. Svetnik V, Wang TC, Ceesay P, et al. Pilot evaluation of a consumer wearable device to assess sleep in a clinical polysomnography trial of suvorexant for treating insomnia in patients with Alzheimer’s disease. J Sleep Res. 2021;30(6)e13328. doi:10.1111/jsr.13328. PMCID: 34340251.

49. Thorpe J, Forchhammer BH, Maier AM. Adapting mobile and wearable technology to provide support and monitoring in rehabilitation for dementia: Feasibility case series. JMIR Form Res. 2019;3(4)e12346. doi:10.2196/12346. PMCID: PMC6913510.

65. Tiersen F, Batey P, Harrison MJC, et al. Smart home sensing and monitoring in households with dementia: User-centered design approach. JMIR Aging. 2021;4(3)e27047. doi:10.2196/27047. PMCID: PMC8387885.

32. van der Wardt V, Hancox JE, Burgon C, Bajwa R, Goldberg S, Harwood RH. Measuring physical activity levels in people with mild cognitive impairment or mild dementia. J Aging Phys Act. 2021;29(1):10-16. doi:10.1123/japa.2019-0234. PMCID: 33049697.

64. Wangmo T, Lipps M, Kressig RW, Ienca M. Ethical concerns with the use of intelligent assistive technology: Findings from a qualitative study with professional stakeholders. BMC Med Ethics. 2019;20(1)98. doi:10.1186/s12910-019-0437-z. PMCID: PMC6924051.

50. Wherton J, Greenhalgh T, Procter R, Shaw S, Shaw J. Wandering as a sociomaterial practice: Extending the theorization of GPS tracking in cognitive impairment. Qual Health Res. 2019;29(3):328-344. doi:10.1177/1049732318798358. PMCID: PMC6380460.
